# Phosphatidylinositol 3-Monophosphate Is Involved in *Toxoplasma* Apicoplast Biogenesis

**DOI:** 10.1371/journal.ppat.1001286

**Published:** 2011-02-17

**Authors:** Lina Tawk, Jean-François Dubremetz, Philippe Montcourrier, Gaëtan Chicanne, Fabrice Merezegue, Véronique Richard, Bernard Payrastre, Markus Meissner, Henri J. Vial, Christian Roy, Kai Wengelnik, Maryse Lebrun

**Affiliations:** 1 UMR 5235 CNRS, Université Montpellier 1 & 2, Montpellier, France; 2 UMR 5237 CNRS, Université Montpellier 1 & 2, Montpellier, France; 3 INSERM U563, Université Toulouse III Paul-Sabatier, CHU Toulouse, Hôpital Purpan, Toulouse, France; 4 Service Commun de Microscopie Electronique, Université de Montpellier 1 & 2, Montpellier, France; 5 Faculty of Biomedical & Life Sciences, Parasitology, Glasgow Biomedical Research Centre, University of Glasgow, Glasgow, Scotland, United Kingdom; Seattle Biomedical Research Institute, United States of America

## Abstract

Apicomplexan parasites cause devastating diseases including malaria and toxoplasmosis. They harbour a plastid-like, non-photosynthetic organelle of algal origin, the apicoplast, which fulfils critical functions for parasite survival. Because of its essential and original metabolic pathways, the apicoplast has become a target for the development of new anti-apicomplexan drugs. Here we show that the lipid phosphatidylinositol 3-monophosphate (PI3P) is involved in apicoplast biogenesis in *Toxoplasma gondii*. In yeast and mammalian cells, PI3P is concentrated on early endosomes and regulates trafficking of endosomal compartments. Imaging of PI3P in *T. gondii* showed that the lipid was associated with the apicoplast and apicoplast protein-shuttling vesicles. Interference with regular PI3P function by over-expression of a PI3P specific binding module in the parasite led to the accumulation of vesicles containing apicoplast peripheral membrane proteins around the apicoplast and, ultimately, to the loss of the organelle. Accordingly, inhibition of the PI3P-synthesising kinase interfered with apicoplast biogenesis. These findings point to an unexpected implication for this ubiquitous lipid and open new perspectives on how nuclear encoded proteins traffic to the apicoplast. This study also highlights the possibility of developing specific pharmacological inhibitors of the parasite PI3-kinase as novel anti-apicomplexan drugs.

## Author Summary

Phosphatidyinositol 3-monophosphate (PI3P) is important for endocytic fusion events in eukaryotic cells. Despite the importance of this lipid in cell biology, its localization and function in apicomplexan parasites has not yet been extensively explored. In this study, we attribute for the first time a role for PI3P in *Toxoplasma* and identify a function different from classical endosomal trafficking. We show that the perturbation of PI3P function in *T. gondii* induced a morphological alteration of vesicles containing proteins destined for the outermost apicoplast membrane, which accumulated abnormally around the organelle, resulting ultimately in the loss of apicoplasts. These findings suggest a new role for PI3P in a vesicular trafficking process necessary for apicoplast biogenesis and provide an attractive model in which PI3P allows the fusion of vesicles containing nuclear-encoded apicoplast proteins with the apicoplast. As the outermost membrane of the apicoplast is originally derived from the endocytic compartment during the ancestral secondary endosymbiosis event, a fascinating question arises about whether apicomplexan parasites have reshaped the classical PI3P-dependent endocytic machinery to target proteins to the apicoplast.

## Introduction

Phosphoinositides are phosphorylated derivatives of the structural membrane lipid phosphatidylinositol and function both as signalling molecules and as compartment specific localization signals for phosphoinositide-binding proteins. PI3-kinases produce derivatives phosphorylated at the D-3 position of the inositol polar head group and have been organised in three classes based on their domain structures, differences in catalytic activities towards distinct substrates, and modes of regulation [1]. Unicellular eukaryotic organisms generally contain only one PI3-kinase belonging to class III, often termed Vps34 after the extensively studied *Saccharomyces cerevisiae* enzyme. Class III PI3-kinases are present in all eukaryotic organisms and are considered as being the conserved ancestral enzymes before the evolution of additional classes occurred in multicellular organisms [2]. Vps34-type enzymes synthesize only phosphatidyinositol 3-monophosphate (PI3P) through phosphorylation of phosphatidylinositol [2], whereas class I and class II kinases are responsible for the synthesis of PI(3,4,5)P_3_ and PI(3,4)P_2_, respectively [3]. PI3P is involved in endosomal trafficking and its function appears to be conserved in eukaryotic organisms spanning from yeast to mammalian cells and plants [4], [5]. PI3P is localized mainly at the cytosolic leaflet of early endosomes and within intraluminal vesicles of multivesicular bodies (MVB) [6]. Mechanisms involved in maintaining the spatial distribution of phosphoinositide pools include the tuned balance of local enzymatic activities of lipid-kinases and lipid-phosphatases as well as the recruitment of proteins that mediate the sequestration or protection of the phosphorylated headgroup from enzymatic degradation [7]. Two main protein domains have been described to bind PI3P: FYVE-domains [8] and PX-domains [9]. PI3P-dependent protein complexes regulate the fusion of endosomal vesicles leading to the generation of MVBs and finally their fusion with the lysosome in mammalian cells and with the vacuole in yeast [6]. At the early endosome, PI3P is found in a complex with Rab5 and Early Endosome Antigen1 (EEA1), and the latter bridges the complex by binding PI3P directly through its FYVE domain and Rab5 through its Rab5-binding domain. PI3P therefore plays a fundamental role in endosomal trafficking to the lysosome/vacuole by serving as the anchoring element of protein complexes.

Here we analysed the function of PI3P in *Toxoplasma gondii*, the causal agent of toxoplasmosis. *Toxoplasma* belongs to the phylum Apicomplexa that includes the malaria parasite *Plasmodium* spp., for which *T. gondii* often serves as a model system in cell biological studies. Apicomplexa are obligate intracellular protozoan parasites. While their compartmental organization resembles the one of a classical eukaryotic cell, they contain in addition a number of specialized organelles, among which a non-photosynthetic plastid termed the apicoplast [10], [11]. This latter is essential for parasite viability and has been acquired by secondary endosymbiosis and in consequence is bounded by four membranes. Concerning endosomal trafficking little is known in *Toxoplasma*. The parasite contains an endomembrane network including an endoplasmic reticulum as well as a single Golgi stack. A Rab5 homologue, TgRab5-1, has been localized to tubovesicular structures adjacent to, but distinct from the Golgi and has been suggested to be involved in cholesterol uptake by the parasite [12]. Interestingly, there is no morphological equivalent to a lysosomal system in *T. gondii* [13]. However, the rhoptries, which are secretory organelles essential for host cell invasion, have been considered to present certain features of MVBs or secretory lysosomes [14]: they have a slightly acidic pH [13] and they contain large quantities of lipids that might be organized in membranes.

In order to explore the function of PI3P in *T. gondii*, we used the regulated expression of a fluorescent PI3P-specific probe to assess the localization of this lipid. We could not detect a link between PI3P and the Rab5-compartment or the rhoptries, but surprisingly we found that PI3P was associated with the apicoplast. Interference with regular PI3P function led to the loss of this essential organelle and our data suggest a role of PI3P in the trafficking of nuclear encoded proteins to the apicoplast. This unexpected finding implicates PI3P in an unusual trafficking pathway that might, in the long term, be exploited as a specific drug target in the fight against apicomplexan parasites.

## Results

### PI3P is synthesized by *T. gondii* tachyzoites

**Figure 1:**
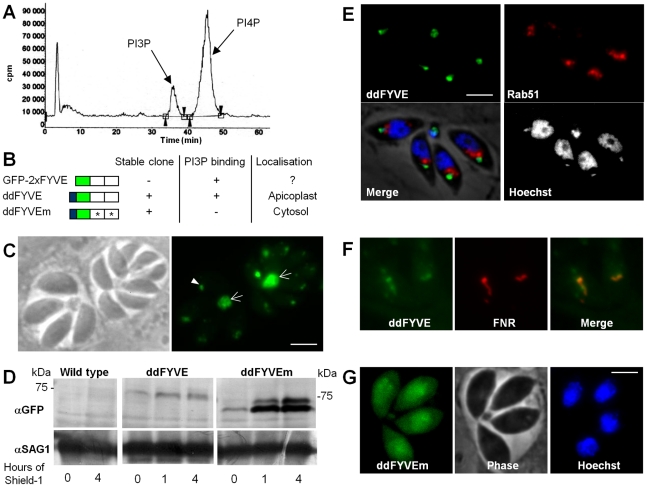
Expression and localization of PI3P in *T. gondii*. (**A**) Extracellular tachyzoites were labelled with [^32^P]orthophosphate, and phosphatidylinositol-monophosphates were analyzed by a combination of thin layer chromatography and HPLC. Retention times of 36 and 45.5 min identified the peaks as PI3P and PI4P, respectively. (**B**) Schematic representation of constructs expressed in *T. gondii*. PI3P-binding capacity of the constructs, the ability to generate stable parasite clones, and the observed localizations are indicated. (**C**) Fluorescence microscopy analysis of parasites transiently transfected with GFP-2xFYVE. Arrowheads indicate the PI3P-enriched compartment at the sub-apical pole of the parasite and arrows point to the accumulation of the FYVE marker in the residual body. Scale bar  = 2 µm. (**D**) Western blot analysis of ddFYVE and ddFYVEm proteins. Intracellular parasites grown with or without Shield-1 (1 µM) were lysed from host cells by passage through a 26-gauge needle and boiled in reducing sample buffer and then separated by SDS PAGE, before probing with anti-GFP. Equal numbers of parasites were loaded in each lane. Time of Shield-1 incubation is indicated. The difference of mobility between ddFYVE and ddFYVEm has also been observed for GST-FYVE and GST-FYVEm recombinant proteins produced in *E. coli* (our unpublished data), and thus appears to be a consequence of these mutations. Anti-SAG1 antibody was used as a loading control. (**E**) ddFYVE expressing parasites were transiently transfected with Rab51-HA and processed for IFA in absence of Shield-1 using the anti-HA antibodies. ddFYVE is enriched in a compartment that is distinct from the one labelled with the endosomal marker Rab51-HA. (**F**) Fluorescence analysis of a parasite clone expressing both ddFYVE and FNR-RFP shows co-localization of both markers in the absence of Shield-1. (**G**) Stable transfected parasites with the mutated construct ddFYVEm show diffuse cytosolic staining after 20 min of Shield-1 induction (fluorescence of ddFYVEm was not observed in the absence of Shield-1). Scale bar  = 2 µm.

The *T. gondii* genome database, ToxoDB [15], features a single putative PI3-kinase (TGME49_015700) belonging to the class III, Vps34–type enzymes [16] as expected in a unicellular eukaryotic organism. To confirm the presence of PI3-kinase activity in *T. gondii*, purified extracellular tachyzoites were metabolically labelled with [^32^P]-orthophosphate and phosphoinositides were extracted and analysed by thin layer chromatography (TLC) and high performance liquid chromatography (HPLC). The phosphoinositide profile revealed the presence of two phosphatidylinositol monophosphates representing PI3P and PI4P (Figure 1A). The amount of PI3P was important representing about a quarter of total phosphatidylinositol monophosphates (23.4% +/−8.6% (SD), n = 3).

Having established that *T. gondii* synthesized PI3P, we next wanted to determine its localization within the parasite. For this purpose, we used a fusion of the green fluorescent protein (GFP) to a tandem repeat of the FYVE domain of the mammalian Hrs protein as a PI3P-specific probe. This construct has been described to specifically bind PI3P [17] and has been used in various organisms to monitor the intracellular localization of PI3P [18], [19]. Parasites were transfected with a construct leading to constitutive expression of the PI3P probe (construct GFP-2xFYVE, Figure 1B) and allowed to invade HFF cells. Fluorescence microscopy performed one day after transfection revealed a sub-apical fluorescent dot in each parasite (Figure 1C). In addition, a strong fluorescent signal was also found in all residual bodies, which are structures left over in the vacuole after parasite division (Figure 1C). *T. gondii* tachyzoites multiply by endodyogeny, a process fundamentally different from division in other eukaryotic cells. Two daughter cells are assembled within the mother cell, ultimately encapsulating most of the mother cell content. Finally the daughter cells derive their plasma membrane from that of the mother cell as they emerge, sometimes leaving behind a small residual body [20], [21]. The dynamics of the sub-apical GFP labelled compartment during cell division could be followed by time lapse fluorescence microscopy. Video S1 and Figure S1 show that GFP-2xFYVE-expressing parasites divided normally and could be used to monitor PI3P localization during parasite development. The sub-apical dot of GFP-2xFYVE moved to the residual body at a discrete step during division corresponding to the separation of the two daughter cells. This event was observed in all dividing transfected parasites and explains the important accumulation of GFP label in the residual body. Concomitantly, new small dots appeared in the nascent daughter cells and ultimately fused to give a larger dot at the sub-apical end of the parasite, indicating that the PI3P-containing compartment was synthesized de novo very early (ten minutes after cell separation) in nascent daughter cells (Figure S1).

However, although we could observe a GFP-2xFYVE signal in transiently transfected parasites, we failed to generate stable transgenic parasites expressing the construct despite performing five independent transfections, suggesting that over-expression of a PI3P-binding domain was disturbing physiological PI3P homeostasis and PI3P-dependent functions and that it was detrimental to the parasite.

### PI3P localization in *T. gondii*

Detailed functional studies could not be conducted with transiently transfected parasites expressing a construct that was not tolerated. We therefore generated transgenic parasites that allowed conditional regulation of GFP-2xFYVE protein levels. Stable clones were obtained with construct ddFYVE (Figure 1B) that expressed GFP-2xFYVE as a C-terminal fusion to the protein-“destabilization domain” (dd) of the FKBP-system [22]. The dd-tagged proteins are systematically and rapidly degraded by the proteasome but can be stabilized upon treatment with the small cell-permeable ligand Shield-1 [23], allowing conditional expression of the protein of interest. As a control we also generated a stable parasite line expressing ddFYVEm, in which both FYVE domains carry a point mutation rendering the fusion protein incapable of binding PI3P [24] (Figure 1B).

**Figure 2:**
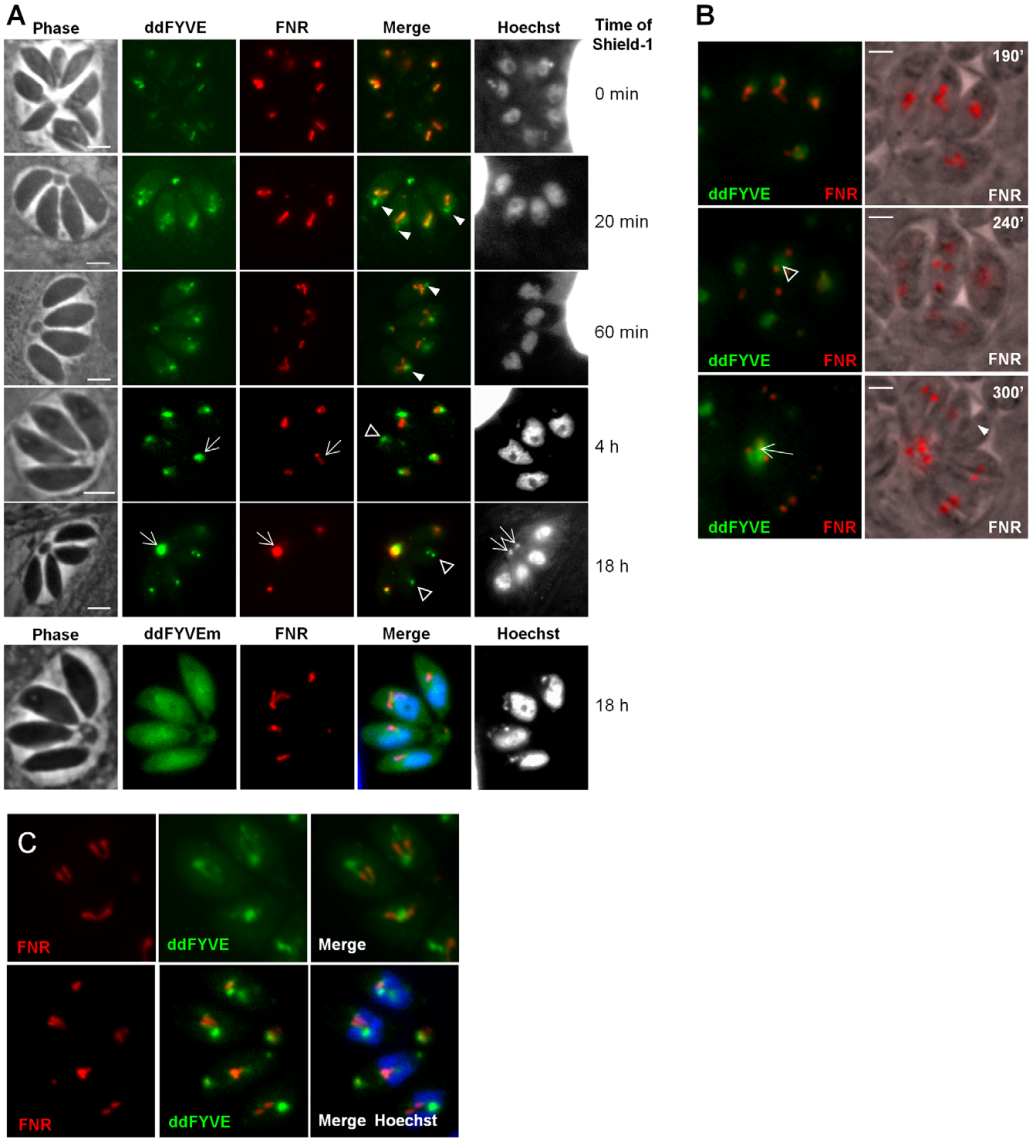
PI3P-binding module disturbs apicoplast biogenesis. (**A**) The expression of ddFYVE in intracellular FNR-RFP/ddFYVE expressing parasites was induced by the addition of 1 µM Shield-1 and the localization of both markers was analyzed at the indicated time of incubation. Arrowheads indicate the formation of a PI3P-containing compartment next to the apicoplast and at the base of V-shaped dividing apicoplasts. DNA-, ddFYVE- and FNR labels in residual bodies (arrows) and tachyzoites that have lost their apicoplast, but retain the PI3P-containing compartment at the sub-apical pole (open triangles) are indicated. The lowest panel shows the cytosolic staining of ddFYVEm and the persistence of the apicoplast 18 h after Shield-1 induction. (**B**) Time-lapse microscopy of FNR-RFP/ddFYVE parasites. Elapsed time after addition of 1 µM Shield-1 is indicated. Early during apicoplast replication, ddFYVE remained concentrated near the apicoplast (190 min). When apicoplasts started to separate (240 min), a portion of FRN label remained associated with the ddFYVE compartment (open triangle). After completion of daughter cell formation (300 min), all ddFYVE label and part of FNR label accumulated in the residual body (arrow). The arrowhead points to the parasite that has lost the FNR label. (**C**) Two hours after Shield-1 induction, the accumulation of ddFYVE is observed at the base of the V-shaped elongated apicoplasts.

Despite the presence of a destabilization domain, a faint ddFYVE signal could already be observed by western blot analysis in the absence of Shield-1 treatment of the parasites (Figure 1D). The signal increased upon addition of the Shield-1 ligand, reaching a maximum after about 1 hour, and remaining stable thereafter (Figure 1D). The level of ddFYVE expression was considerably lower than that of the mutated version ddFYVEm, suggesting that it would be less tolerated by the parasite and might be degraded. IFA analysis detected ddFYVE in the absence of Shield-1 as a dot in the sub-apical part of the parasite (Figure 1E), similar to the one described above with transient transfection (Figure 1C). In other organisms PI3P is concentrated on early endosomes and co-localizes with Rab5 [17]. In *T. gondii*, however, we found that ddFYVE did not co-localize with the early endosome marker TgRab5.1 [12] (Figure 1E), but most unexpectedly co-localized instead with the apicoplast, whose DNA can be stained and appears as a small dot next to the nucleus (Figure 1E). This was subsequently confirmed by co-localization of ddFYVE with the FRN-RFP apicoplast marker [25] transfected in the ddFYVE strain (Figure 1F). Shield-1 treatment induced a progressive increase in fluorescence and some ddFYVE staining also appeared to surround the apicoplast (Figure 2A, see below). In striking contrast, the mutated form ddFYVEm was found throughout the cytoplasm (Figure 1G), confirming that the specific localization of the native construct was due to functional PI3P-binding domains. To confirm PI3P localization in *T. gondii*, we probed infected cells with anti-PI3P antibody and observed in each parasite a bright dot of fluorescence that partially co-localized with apicoplast (Figure S2). We therefore concluded that PI3P-enriched compartments are localized at the apicoplast and in its vicinity.

### Over-expression of an exogenous PI3P-binding domain interfered with cellular functions necessary for apicoplast biogenesis

We then performed a time-course analysis of the fate of the ddFYVE strain incubated with Shield-1. Interestingly, this experiment revealed that the localization of ddFYVE varied with time. From 20 min onwards, the GFP signal became more and more diffuse around the apicoplast (Figure 2A). A quantitative analysis of co-localization between ddFYVE-GFP and FNR-RFP by defining Manders co-localization coefficients on Z-sections showed a decrease of co-localization from 0.7±0.08 to 0.31±0.1 between time 0 and 4 h after Shield-1 treatment (Figure S3). Most surprisingly, prolonged expression of ddFYVE induced the loss of the FNR-RFP signal in a variable number of parasites within a given vacuole (Figure 2A). Concomitantly, both FNR-RFP and ddFYVE labels were detected in the residual body, indicating severe disturbance of this stromal apicoplast protein marker. Hoechst-staining confirmed the absence of apicoplast DNA in some Shield-1 treated parasites and its presence in the residual body in these vacuoles, suggesting that “lost” apicoplasts had ended up in this structure (Figure 2A, 18 h). At the same time, a sub-apical PI3P-rich compartment was still detected in apicoplast-defective parasites (Figure 2A, 18 h). The deleterious effect of ddFYVE expression on the apicoplast was specific for this organelle. Other cellular compartments like the micronemes, rhoptries, mitochondrion, Golgi, and early endosomes were not disturbed (Figure S4). Notably, even after prolonged Shield-1 induction, we did not observe co-localization between Rab5.1 and ddFYVE (Figure S4). Moreover, no deleterious effect on the apicoplast was observed after prolonged expression of the mutated form ddFYVEm (Figure 2A) demonstrating that the defect was specifically linked to the binding of PI3P by ddFYVE. Apicoplast loss upon ddFYVE expression was not observed in all dividing parasites. This could be due to the fact that the expression levels of ddFYVE may vary between cells with the dd system [22] and would reinforce the idea that the extent of the phenotype would be dependent on the amount of ddFYVE present to interact with PI3P. In fact, quantification of FNR-RFP expressing parasites showed that 24 h after invasion and culture in the presence of Shield-1, 15.5%±0.8% of ddFYVE expressing parasites had lost their apicoplast, and this defect increased to 31.9%±2.8% after an additional 48 h pre-incubation with Shield-1. As a control, only 1% of mock treated parasites showed detectable loss of the FNR-RFP apicoplast marker (data not shown).

Expression of a fluorescent FYVE-construct allows imaging PI3P location in live cells with the caveat that competition of the marker with endogenous PI3P-binding proteins (PI3P effectors) may alter the normal function of the inositide [26]. In the present case however, low level expression of ddFYVE in the absence of Shield-1 allowed imaging the PI3P-enriched compartment with minimal disturbance, whereas high level ddFYVE expression after prolonged Shield-1 treatment induced disruption of PI3P function by sequestration of the lipid. Double fluorescent ddFYVE/FRN-RFP parasites were therefore used to follow the fate of the apicoplast FNR-RFP marker by time lapse fluorescent microscopy during cell division in the presence of the disturbing PI3P-binding module (Figure 2B and Videos S2 and S3). During endodyogeny, apicoplast division is tightly associated with nuclear- and cell division and is characterized by an elongated, V-shaped intermediate, with both ends linked to the centrosomes [25], that eventually splits through a process driven by a dynamin-related protein [27]. Two hours after ddFYVE induction, PI3P was concentrated at one end or at the base of V-shaped elongating apicoplasts (Figure 2C). In some parasites, when the apicoplasts separated, the FNR label remained associated with the ddFYVE labelled compartment (Figure 2B, middle panel) and ultimately collapsed together with the totality of ddFYVE into the residual body when the two daughter cells separated. Videos S2and S3 show the disappearance of one apicoplast during the transition from four to eight parasites. Thus, sequestration of PI3P during endodyogeny resulted in apicoplast-deprived daughter tachyzoites.

### Depletion of parasite PI3P by inhibition of the PI3-kinase activity disturbs apicoplast biogenesis

**Figure 3:**
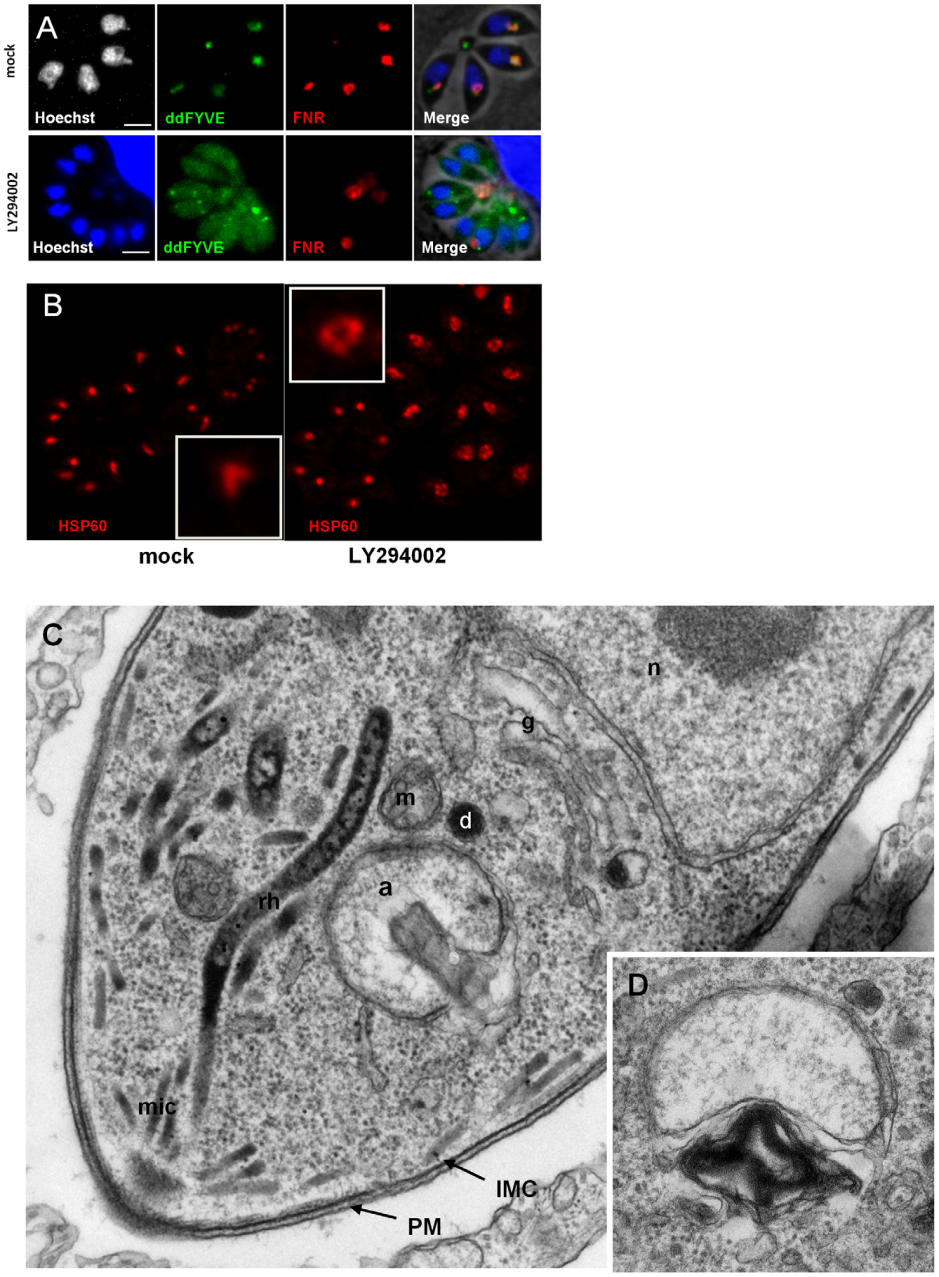
The PI3-kinase inhibitor LY294002 interferes with apicoplast biogenesis. (**A**) ddFYVE/FRN-RFP expressing parasites were treated with 100 µM LY294002 for 4 h or mock treated before detection of PI3P by ddFYVE stabilization for 20 min with 1 µM Shield-1. Note the efficient depletion of PI3P by the inhibitor, revealed by the cytosolic localization of ddFYVE. Seven of the eight parasites have lost their apicoplast while FNR- and DNA label accumulate in the residual body. Scale bar  = 2 µm. (**B**) Immunofluorescence analysis of a parasite treated with 50 µM LY294002 for 4 h using antibodies against the stromal protein Hsp60 shows swollen apicoplasts. (**C, D**) EM analysis of wild-type parasites fixed 4 hours after addition of 100 µM LY294002. (C). The parasite ultrastructure is not altered, except at the level of the apicoplast, which displays abnormal membrane whorls and myelinic profiles accumulating under the outer membrane of the organelle. a, apicoplast; IMC, inner membrane complex; m, mitochondrion; n, nucleus; PM, parasite plasma membrane; mic, micronemes; rh, rhoptrie; g, Golgi. (D) Accumulation of membranous whorls in the outer membranes of the apicoplast. Bar  = 0.5 µm.

To confirm the implication of PI3P in apicoplast biogenesis, we used an alternative strategy by inhibiting the PI3P-producing enzyme PI3-kinase. LY294002 and wortmannin are well established inhibitors of the PI3-kinase [28]. LY294002 showing the advantage of substantially higher stability in complex culture media, was used throughout the study. Intracellular ddFYVE- and FNR-RFP co-expressing parasites were treated with 100 µM LY294002 for four hours and depletion of PI3P as a consequence of PI3-kinase inhibition was evaluated by adding Shield-1 for 20 min at the end of the drug treatment. In comparison to mock-treated controls, LY294002 reduced the prominent subapical ddFYVE dot in *T. gondii* tachyzoites and led to diffuse labelling throughout the cytoplasm, reflecting efficient depletion of PI3P (Figure 3A). Depletion was also observed to a lesser extent at 50 and 25 µM (data not shown). Concomitantly, the localization of the FNR-RFP marker was disturbed and in many vacuoles most of it was now found in the residual body (Fig. 3A) while other cellular markers appeared not affected (Figure S5). Apicoplast loss was not significantly observed at dilutions below 100 µM LY294002 (Figure S6) but the apicoplast appeared rounder (Figure 3B) and the number of parasites showing a typical V-shaped elongated apicoplast was reduced (Figure S6), a morphological change that was not significantly observed in ddFYVE expressing parasites. Because 100 µM LY294002 is a high concentration compared to what is normally used on mammalian cells, we evaluated the structural integrity of LY294002 treated parasites by electron microscopy. As shown in Figure 3C, after 4 hours of 100 µM LY294002, the parasites displayed a normal organization, except that the apicoplast appeared misshaped with prominent internal myelinic profiles, reflecting membranous disorder (Figure 3C, D). In addition, and confirming the FNR-RFP observations, discarding of apicoplasts in the residual body was a common feature in LY294002 treated parasites (Figure S7). Taken together, these results strongly indicate that in *T. gondii*, PI3P is involved in cellular functions necessary for apicoplast biogenesis and inheritance in daughter cells.

### ddFYVE expression induced a delayed death phenotype

The phenotype linked to apicoplast loss has been termed “delayed death” and is classically observed either upon drug treatments targeting apicoplast metabolism [29], [30] or as a consequence of apicoplast biogenesis defects [31]. Apicoplast-devoid parasites can invade new host cells and can start replicating, but then stop dividing and die.

**Figure 4:**
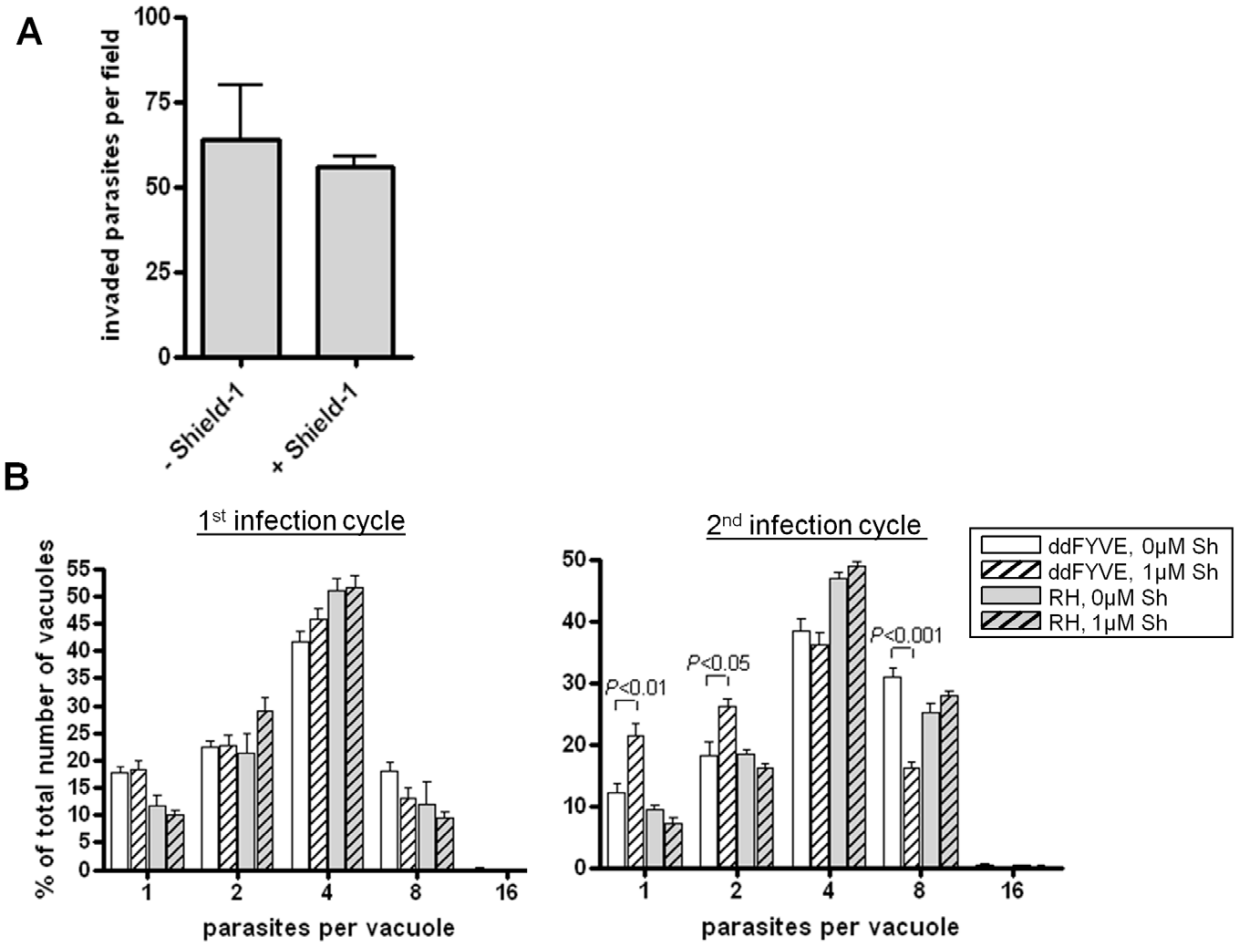
Delayed death phenotype of parasites expressing ddFYVE. (**A**) Expression of ddFYVE does not reduce invasion of HFF cells. Confluent monolayers of HFF cells were incubated with tachyzoites previously incubated with or without Shield-1 for 48 h and invasion was assessed as described in materials and methods. Results are presented as invaded tachyzoite numbers per field. Values are the mean ± SEM (10 fields per assay, n = 4). (**B**) Expression of ddFYVE reduced intracellular growth after reinvasion. The effect of expression of ddFYVE on intracellular growth was measured (A) after 24 h of growth in presence or not of Shield-1, (B) on parasites pre-incubated intracellularly for 2 days in the presence or absence of Shield-1, then purified and allowed to reinvade HFF for 24 h. The number of vacuoles containing only one or two parasites was statistically higher for parasites having expressed ddFYVE during the previous cycle (*P*<0.01 for one parasite per vacuole and *P*<0.05 for two parasites per vacuole). Conversely, the number of vacuoles containing eight parasites was statistically lower as compared to wild type or to untreated ddFYVE transfectants (*P*<0.001). No effect was observed with untransfected (RH) parasites. Values are the mean ± SEM (100 vacuoles were counted per assay, n = 4) of one representative experiment. Graphs and statistical analysis were done with GraphPad Prism. Two-tailed *P* values were determined by unpaired t-test.

We thus evaluated whether ddFYVE expression would induce a delayed death phenotype. We first compared the number of parasites per vacuole 18 h after infection between ddFYVE expressing parasites grown in the presence of Shield-1 and parasites grown without drug. No significant reduction in parasite proliferation was found (Figure 4B). Then, intracellular ddFYVE parasites were treated or not with Shield-1 for 48 h and mechanically released from host cells before adding them to new host cells. We found no significant difference in the ability of untreated tachyzoites versus ddFYVE-expressing parasites to invade host cells (Figure 4A), showing that the invasion process was not affected. In contrast, pre-incubation of intracellular parasites with Shield-1 for 48 h before mechanical release and reinvasion of fresh cells followed by counting the number of parasites 24 h after invasion demonstrated a reduction in parasite growth of tachyzoites that had been grown in the presence of Shield-1 during the previous intracellular cycle (Figure 4B). The number of vacuoles containing one or two parasites was significantly higher for ddFYVE-expressing parasites grown in presence of Shield-1 while, conversely, the number of vacuoles containing eight parasites was significantly lower. Thus, the expression of a PI3P-binding module induced a delayed intracellular growth defect without affecting invasion as expected for apicoplast deprived parasites. This result also offered an explanation for our failure to generate and maintain stable parasite lines constitutively expressing high levels of GFP-2xFYVE.

### PI3P is associated with the apicoplast and neighboring vesicles

**Figure 5:**
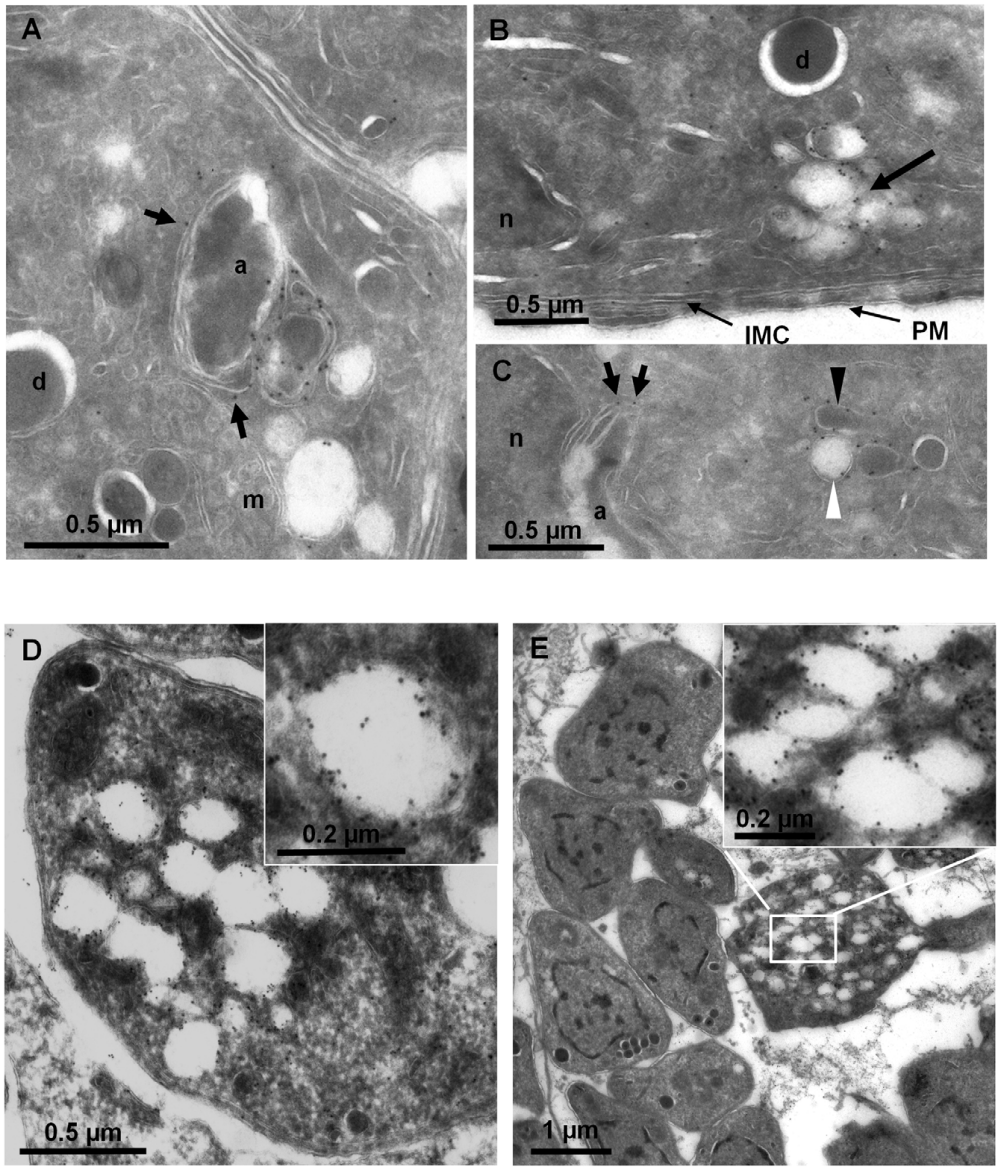
Localization of PI3P in *T. gondii*. ImmunoEM analysis of ddFYVE expressing parasites fixed 20 min (A, B, C) or 24 h (D, E) after addition of 1 µM Shield-1. (**A, B, C**) The anti-GFP gold label is found on the outer membranes of the apicoplast (short arrows), on the membrane of electron-dense (C, black arrowheads) and electron-lucent (C, B, white arrowheads) vesicles, these latter accumulating in the subapical region (B, arrow). a, apicoplast; d, dense granule; IMC, inner membrane complex; m, mitochondrion; n, nucleus; PM, parasite plasma membrane. (**D, E**) After one day of Shield-1 treatment, the anti-GFP labels large lucent vacuoles either inside parasites (D), or accumulated in the residual body (E), while the overall structure of the tachyzoites is unaffected. The white framed boxes show enlargements of GFP-positive lucent vesicles.

In order to further refine the localisation of PI3P, the ddFYVE signal was analyzed by immuno-electron microscopy (IEM). Due to the low level of spontaneous expression of ddFYVE, no signal could be detected in IEM in the absence of Shield-1. Experiments were therefore performed within the first hour of Shield-1 addition, at a time when the PI3P signal started diffusing around the apicoplast but before the loss of the apicoplast could occur (Figure 2A). Anti-GFP antibodies labelled the outer membranes of the apicoplast and the membrane of vesicles located in the vicinity of this organelle (Figure 5A, B, C). The vesicles were packed together and were of variable electron density: the denser ones were of uniform size, usually spherical with a diameter of 0.1±0.01 µm; the electron-lucent ones were larger, more heterogeneous in size and shape (usually ovally shaped, 0.18±0.07×0.22±0.1 µm). In parasites fixed 24 h after Shield-1 induction (Figure 5D, E), larger clusters of the electron lucent PI3P-vesicles were found, and these vesicles also massively accumulated in residual bodies (Figure 5E). In the absence of Shield-1 treatment, some electron-dense vesicles were detected around the apicoplast ([32], [33], [34] and this study), while we could not detect electron-lucent vesicles near the organelle (not shown), suggesting that their presence resulted from disturbing PI3P function.

These results showed that PI3P was associated with the apicoplast membranes and with electron-dense and -lucent vesicles the latter of which appearing and accumulating in the cytosol possibly as a consequence of interference with their fusion to the apicoplast. This finding, together with the disturbance in apicoplast genesis suggested a role of PI3P in vesicular traffic to the apicoplast.

### Over-expression of an exogenous PI3P-binding domain disturbs the vesicles containing outer membrane apicoplast proteins and their traffic to the apicoplast

Our results suggesting a contribution of PI3P in apicoplast biogenesis, we next wanted to elucidate more precisely the part played by PI3P in this process. Most apicoplast proteins are encoded by nuclear genes and must be targeted to the apicoplast, which is bounded by four membranes [10], [11]. They are either imported in the stroma after crossing four membranes or reach different membranes and/or inter-membrane spaces within the apicoplast. The traffic of nuclear-encoded proteins to the apicoplast requires entry into the secretory system (ER) before trafficking, a step that does not occur with double membrane primary plastids, in which proteins are typically synthesized in the cytosol and routed directly into the organelle. However, how these ER-transiting proteins are targeted to the apicoplast outermost membrane is unknown. Recent immuno-electron microscopy studies of proteins associated with outermost apicoplast membranes showed labeling not only of the peripheral compartments of the apicoplast, but also of dense vesicles next to the organelle [32], [33], [34], suggesting a vesicular route to the apicoplast for these peripheral membranes proteins.

**Figure 6:**
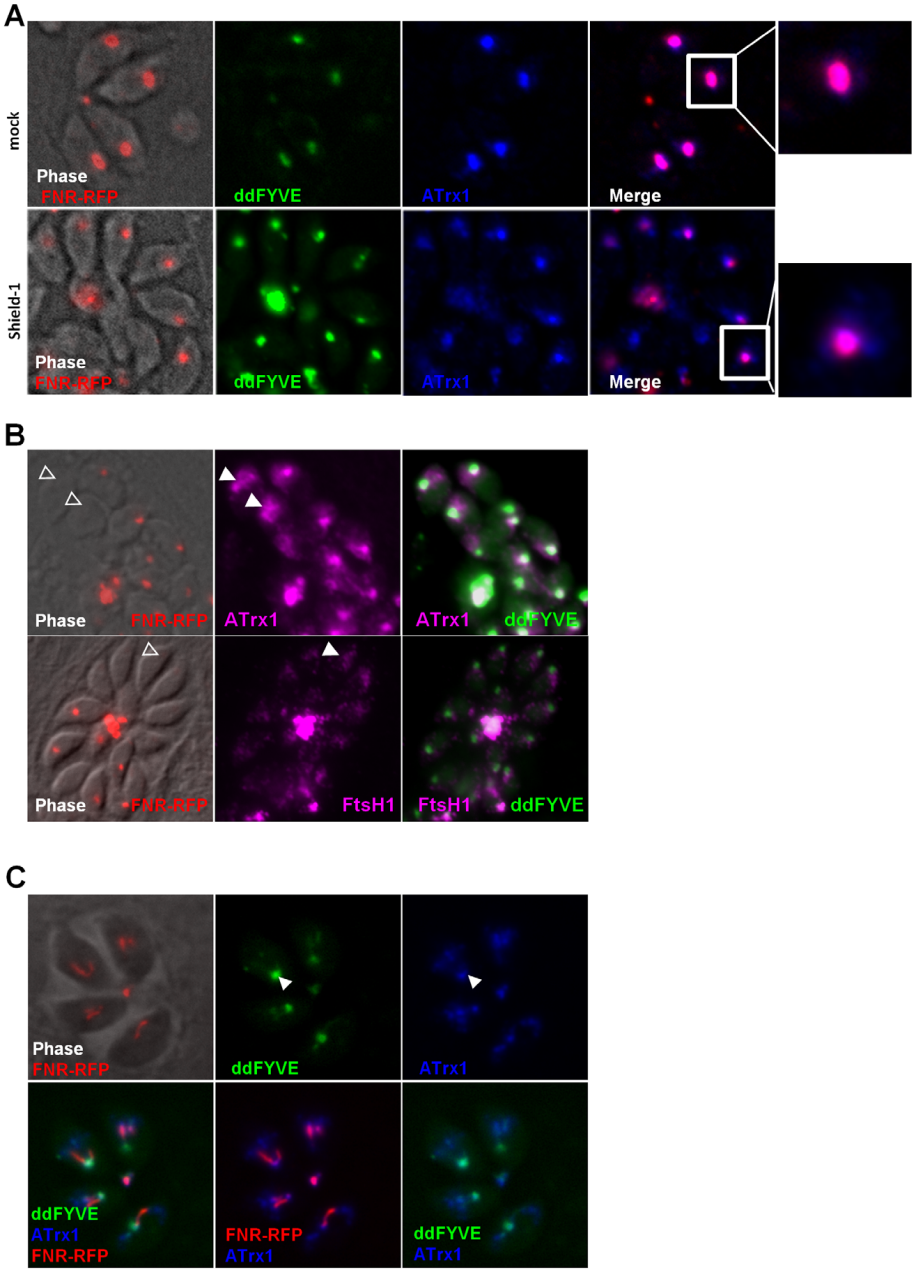
Traffic of apicoplast proteins in the presence of ddFYVE. (**A**) The traffic of outermost apicoplast proteins is disturbed in the presence of ddFYVE. Immunofluorescence analysis of ddFYVE transfected parasites treated for 24 h with Shield-1 revealed a more diffuse labelling of ATrx1 (blue) around the apicoplast FNR-RFP dot (red) after Shield-1 treatment compared to untreated parasites (enlarged in the white framed box of the ATrx1 and FNR-RFP merged image). Single plane apotome sections are shown. (**B**) PI3P-containing vesicles and outermost apicoplast membrane proteins are still detected in the absence of the apicoplast. Open triangles indicate a diffuse staining of membranous ATrx1 (magenta) or FtsH1 (magenta) in FNR-RFP negative parasites. (**C**) Both ddFYVE (green) and ATrx1 (blue) accumulate and co-localize at the base of V-shaped dividing apicoplasts (FNR-RFP, red) in parasites treated for 6 h with Shield-1.

We thus analysed the distribution of the three apicoplast outermost membrane proteins FtsH1 [33], APT1 [34] and the thioredoxin ATrx1 [32] following disturbance of PI3P function. For this purpose, ddFYVE expressing parasites were stably transfected with plasmids coding for V5-FtsH1 or APT1-HA, and subsequently transiently transfected with FNR-RFP and the cells were then treated or not with Shield-1. IFA analysis of FtsH1, APT1 and ATrx1 was performed using anti-V5, anti-HA and anti-ATrx1 (Mab 11G8) antibodies respectively. As described previously, the localization of outermost apicoplast proteins varies during the cell cycle ([35], data not shown). During plastid elongation, these apicoplast proteins localized around the length of the elongated apicoplast as well as on vesicles or tubules nearby, while during the interphase, the label was restricted to the plastid, co-localizing with the FNR-RFP marker. After one day of Shield-1 disturbance of PI3P function, we observed a diffuse staining of FtsH1, APT1 and ATrx1 around the organelle still labelled by FNR-RFP (Figure 6A and Figure S8), suggesting that the fusion of the vesicles containing membranous proteins with the apicoplast was inhibited. Moreover, when the apicoplast had disappeared from some parasites as a consequence of Shield-1 treatment (as detected by the loss of the FNR-RFP marker), the vesicular staining of outermost apicoplast proteins was still detected in all apicoplast-deprived parasites (Figure 6B and Figure S8).

**Figure 7:**
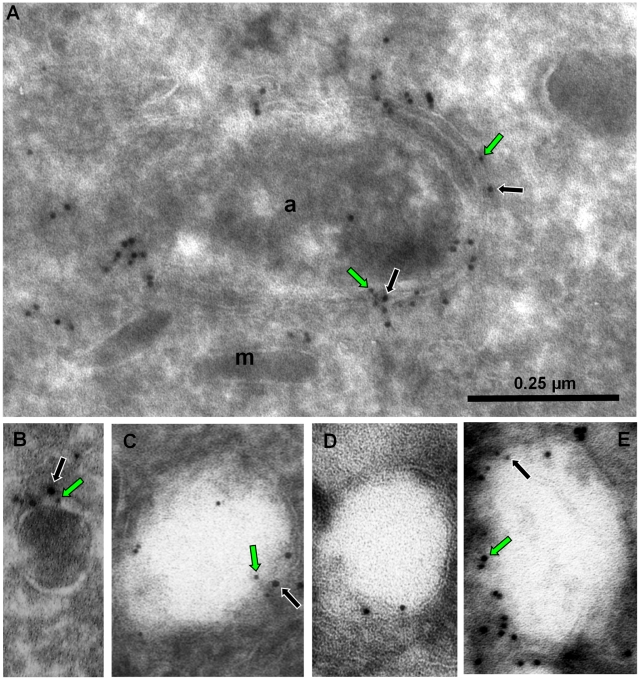
Electron-lucent vesicles accumulate upon ddFYVE expression and contain apicoplast peripheral membrane proteins and PI3P. Immuno-EM analysis of ddFYVE expressing parasites transiently expressing V5-FtsH1 (A, B, C) or APT1-HA (D, E) fixed 4 h (A, B, C) or 24 h (D, E) after 1 µM Shield-1 addition. The scale is the same for all five images. (**A, B, C**) Double immuno-EM with anti-GFP (5 nm gold, green arrows) and anti-V5 (10 nm gold, black arrows) antibodies (a: apicoplast; m: microneme). (A) shows double labelling of the apicoplast outer membranes, (B) of a small dense vesicle, (C) of an electron lucent vesicle. (**D**) Immuno-EM with anti-HA antibodies. (**E**) Double immuno-EM with anti-GFP (10 nm gold, green arrows) and anti-HA (5 nm gold, black arrows) antibodies.

On dividing apicoplasts, we detected an accumulation of ATrx1 at the base of the V-shaped intermediates that co-localized with ddFYVE label (Figure 6C). Double IEM analysis was then conducted on ddFYVE/FtsH1 or ddFYVE/APT1 parasites to localize outermost apicoplast proteins and PI3P simultaneously. After four hours of Shield-1 treatment, anti-V5 and anti-GFP antibodies showed the simultaneous presence of FtsH1 (black arrows) and PI3P (green arrows) on both the outermost membrane of the apicoplast and on dense-core vesicles of ca. 0.1 µm in its vicinity (Figure 7A, B, C), as in untreated control cells ([33] and data not shown) demonstrating that PI3P was present on vesicles containing outermost apicoplast membrane proteins. In addition, we also detected FtsH1 on the larger, ddFYVE positive lucent vesicles. After one day of Shield-1 treatment, most of the vesicles containing APT1 (black arrows) (Figure 6D) and FtsH1 (data not shown) were the large electron lucent vesicles, also shown to be positive for the ddFYVE marker (green arrows) by double IEM (Figure 7E).

Taken together, these results indicated that the disturbance of PI3P function in *T. gondii* induced an abnormal vesicular traffic to the apicoplast.

## Discussion

PI3P, the product of class III PI3-kinase, is important for regulating signalling and trafficking events in eukaryotic cells. It serves as a lipid marker helping the recruitment of FYVE- or PX-domain-containing PI3P effectors, which influence endocytic membrane fusion. In this study, we have developed an approach based on the inducible expression of a PI3P-binding module that disturbed PI3P function in *T. gondii* possibly through competition with endogenous PI3P-binding proteins. As a consequence, vesicles accumulated around the apicoplast, ultimately resulting in the elimination of apicoplasts. Based on these results, we attribute for the first time a role for PI3P in *T. gondii*, which in addition is different from classical endosomal traffic. Our results suggest a role for PI3P in a vesicular traffic to the apicoplast that appears to be an essential step in apicoplast biogenesis. This study supports an attractive model, in which PI3P allows the fusion of vesicles containing nuclear-encoded apicoplast proteins with the outermost membrane of the apicoplast.

### Development of a conditional tool to image PI3P and inhibit PI3P function

Estimating the distribution of phosphoinositides in a cell is a challenging endeavour. The most common approaches are either by analytical assays on isolated membrane fractions or through the use of specific probes. Since phosphoinositides are often present in very small amounts, and cellular fractionation of intracellular parasites is a difficult task, imaging phosphoinositides using specific probes constructed from phosphoinositide-binding domains is the tool of choice. Here, we have deciphered both the localization of PI3P and its function in *T. gondii* by expressing a fluorescent GFP-FYVE probe in the parasites. Constitutive expression of the corresponding transgene was deleterious for *T. gondii*, indicating that interference with PI3P function was not tolerated and therefore that PI3P is necessary for parasite survival. PI3P exerts its function through the binding of effector proteins that contain PI3P binding domains. Expression of an exogenous PI3P-binding module probably sequesters the lipid and thus exerts a dominant negative effect on the functions of endogenous PI3P-binding proteins. Imaging live cells with fluorescent probes is a useful method, but its drawback is that it could alter normal functions and thereby subcellular localization of the targeted molecule [26]. Here, we circumvented this problem by generating transgenic parasites that allowed regulation of FYVE protein levels through fusion to the protein-“destabilization domain” (dd) of the FKBP-system [22]. However, and in this case luckily, even in the absence of Shield-1 a small amount of ddFYVE protein was stable but had no effect on organelle morphology (Figure S4), invasion and intracellular growth (Figure 4). This led us to localize the lipid to the apicoplast and its vicinity in the absence of any detectable deleterious effect. This localization was also confirmed using anti-PI3P antibodies. Only upon extended over-expression of the exogenous PI3P-binding module did we observe, as upon constitutive expression of GFP-2xFYVE, the loss of apicoplasts and the inhibition of intracellular growth after reinvasion of apicoplast-deprived parasites. Conditional expression of ddFYVE therefore proved to be a very useful approach both to image the PI3P-enriched compartment under unperturbed conditions (no Shield-1) and to disturb PI3P function by sequestration of this lipid (upon Shield-1 induction).

### A potential PI3P-dependent vesicular route from the ER to the apicoplast for the delivery of apicoplast peripheral membrane proteins

We have identified the apicoplast and surrounding vesicles as the major PI3P-containing subcellular compartments in *T. gondii*. It should be noted that our approach did not allow access to PI3P already engaged with an effector protein or within the lumen of intracellular compartments such as for example the rhoptries, which contain large quantities of lipids that might be organized as membranes [36] or such as the three inner apicoplast membranes. PI3P was detected on the apicoplast membrane and became mainly associated with vesicles around the apicoplast upon extended Shield-1 incubation. IEM analysis showed that the content of these vesicles varied from electron-dense to electron-lucent, with intermediate stages, the lucent becoming especially abundant after prolonged Shield-1 incubation. Although mature *Toxoplasma* parasites contain numerous vesicular structures, the group of GFP-FYVE labelled electron-lucent vesicles next to the apicoplast was not observed in wild type or untreated parasites, suggesting that these accumulating vesicles could not fuse with the apicoplast and highlighting the possible implication of PI3P in a vesicular traffic to the apicoplast.

Apicoplast proteins are either imported in the stroma after crossing four membranes or reach different membranes and/or inter-membrane spaces within the apicoplast. Recent immuno-electron microscopy studies of proteins associated with outermost apicoplast membranes showed labeling not only of the peripheral compartments of the apicoplast, but also of dense vesicles next to the organelle [32], [33], [34]. Our results suggest that PI3P is involved in the traffic of these vesicles to the apicoplast. Indeed, we show that they contain both PI3P and apicoplast peripheral membrane proteins. Moreover, perturbation of PI3P signaling induces both the accumulation of these vesicles around the apicoplast and their morphological changes. While they are exclusively dense core vesicles under normal conditions ([32], [33], [34] and this study), they become electron-lucent and increase in size when ddFYVE is over-expressed. Intermediate density vesicles are also found, strongly suggesting that the abnormal fate of these peripheral apicoplast protein loaded vesicles induces a dilution of their contents. Even though it remains to be elucidated how these alterations occur, these observations indicate a specific disturbance of vesicles containing apicoplast peripheral membrane proteins after PI3P sequestration. The accumulating vesicles are unlikely to arise from apicoplast disruption because no significant alteration of the apicoplast was observed after ddFYVE expression, but are rather a consequence of the accumulation of a transient pool of vesicles normally destined to fuse with the organelle. Therefore, our observations suggest a role of PI3P in the fusion of endoplasmic reticulum derived transition vesicles carrying nuclear-encoded proteins to the outermost apicoplast membrane. We propose that PI3P serves as a marker to recruit PI3P-binding proteins that promote fusion of these vesicles with the apicoplast. Expression of an exogenous FYVE module in *T. gondii* would then sequester free PI3P and inhibit the binding of endogenous FYVE- or PX-containing proteins. Six putative PI3P-binding proteins (i.e, containing PX- or FYVE domains) are predicted in ToxoDB [15]. Interestingly, we detected the presence of a *T. gondii* FYVE containing protein in the vicinity of the apicoplast (unpublished results). This protein co-localized with the ddFYVE marker after Shield-1 induction, suggesting that it could participate in the formation of the complex interaction network involved in the fusion of the vesicles with the apicoplast.

Our study shows that, after perturbing PI3P function by over-expression of ddFYVE, the vesicles containing peripheral membrane proteins are still present even though they are progressively altered and there is no apparent accumulation of these proteins at the ER, indicating that PI3P exerts its function downstream of their formation. When we inhibit the production of PI3P by inhibition of the PI3-kinase, a far more drastic and rapid effect on the apicoplast membranes is observed before any alteration of the vesicles, as if PI3P was not only involved in trafficking of proteins to the organelle, but also in the homeostasis of the organelle itself and regulation of its membrane lipid content. The role played by PI3P in vesicular trafficking to the apicoplast therefore needs further analysis through more specific tools such as the generation of an inducible PI3-kinase gene knock-out and the characterization of the PI3P effectors *in T. gondii*.

### Endosomal trafficking in apicomplexan parasites

In eukaryotic organisms spanning from yeast to mammalian cells and plants PI3P is involved in endosomal trafficking. PI3P is principally produced at the Golgi apparatus and early endosomes and plays a crucial role as recognition marker for binding the Rab5 GTPase and EEA1, a protein with a FYVE domain. The latter interacts with SNARE proteins and promotes vesicular fusion of endocytic vesicles. Specific depletion of PI3P in the endosomal compartment affects yeast vacuole morphology and disrupts the normal maturation of the endosomal compartment [37]. In addition, inhibition of PI3-kinase with wortmannin prevents fusion of the phagosome with late endosomes [38].

Endosomal trafficking in apicomplexan parasites is poorly known except for the vesicular transport of haemoglobin in *Plasmodium* blood stage parasites that internalize host haemoglobin, which is subsequently degraded in the food vacuole. In transgenic *P. falciparum* parasites that expressed a constitutively active mutant of Rab5a, the protein localized to small haemoglobin containing vesicles [39]. A recent study in *Plasmodium* showed that PfPI3-kinase could be involved in haemoglobin endocytosis and trafficking in the parasite [40]. Treatment of *Plasmodium* cultures with PI3-kinase inhibitors induced the entrapment of haemoglobin vesicles in the parasite cytosol and prevented their fusion with the food vacuole. We could recently show through expression of a GFP-2xFYVE construct in *P. falciparum* that PI3P localized to the food vacuole membrane [41] further supporting a role of PI3P in endocytosis and traffic to the food vacuole.

In *Toxoplasma*, there is no food vacuole and the endosomal system is still poorly characterized. We could not detect any link between PI3P and Rab5-positive vesicles in the parasite. Also, the morphology of the rhoptries, the only organelle described so far to share certain common features with a lysosomal compartment in *Toxoplasma*, appeared unaltered by the expression of GFP-FYVE. In contrast our data clearly point to a role of PI3P in vesicular traffic towards a distinct organelle, the apicoplast. Interestingly, in *P. falciparum* in addition to the food vacuole, we also observed by immunoelectron microscopy the presence of dense and lucent vesicles bearing PI3P close to the apicoplast, which was itself labelled [41]. This suggests that a PI3P-dependent trafficking of peripheral apicoplast membrane proteins is likely to be conserved in apicomplexan parasites.

The apicoplast is derived from a secondary endosymbiosis of a red alga that had itself internalized a cyanobacterium-like prokaryote. The apicoplast is bounded by four membranes [10], [11]. The inner two membranes are thought to derive from the first endosymbiosis event and hence to be of cyanobacterial origin. In contrast, the outermost two membranes are thought to derive from the secondary endosymbiosis event. One originates from the plasma membrane of the engulfed algal cell; the periplastid membrane. The outermost membrane of the apicoplast is analogous to the phagosomal membrane of the host cell; hence it is of endosomal origin. It has been hypothesized that the protein-import machinery of each apicoplast membrane reflects the origin of that membrane [27]. As the outermost membrane of the apicoplast is originally derived from the phagocytic compartment during the secondary endosymbiosis event, the fascinating question arises whether apicomplexan parasites have reshaped the classical PI3P-dependent endocytic machinery found in other eukaryotes to target proteins to the apicoplast.

## Materials and Methods

### Parasite culture

Tachyzoites of the RH *hxgprt-* strain of *T. gondii* deleted for hypoxanthine guanine phosphoribosyl transferase (ΔHX strain) [42] were used throughout the study. All *T. gondii* tachyzoites were grown in human foreskin fibroblasts (HFF) or Vero cells in standard condition. Shield-1 (ClonTech) and LY294002 (Sigma) were used at 1 µM and 100 µM respectively, unless otherwise stated.

### PI3P extraction and analysis

Extracellular *T. gondii* RH tachyzoites (1.5×10^9^) were separated from host cells by filtration [43], washed twice with 137 mM NaCl, 4 mM KCl, 1 mM MgCl_2_, 1 mM CaCl_2_, 10 mM glucose, 30 mM Hepes pH 7.4, and labelled with 500 µCi [^32^P]H_3_PO_4_ for 1 h at 37°C. Phosphoinositides were extracted and analysed as described [44].

### Plasmid constructs

The *Nco*I/*Sma*I GFP-2xFYVE fragment of pEGFP2xFYVE [17] was cloned in *Nco*I/*Nsi*I-blunted pTUB8mycGFPPftailTY to generate pGFP-2xFYVE. For plasmid p-ddFYVE, mycYFP of pTUB-DDmycYFP-CAT [22] was replaced as an *Avr*II/*Not*I fragment by GFP-2xFYVE, the latter being PCR amplified from pGFP-2xFYVE with primers 5′-ATTCCTAGGATGGTGAGCAAGGGCGAGG-3′ and 5′-TGTTCTGGCAGGCTACAGTG-3′. The double C^215^S mutated 2xFYVEm was PCR amplified from plasmid pGST-2xFYVEm [17] with primers 5′-AAGCTTCTCGAGCCGGAATTCGAAAGTG-3′ and 5′-GGATCCTTAAGCTCGACTCGACTTATGC-3′ and cloned as *Hind*III/*Kpn*I fragment in ddFYVE, yielding plasmid p-ddFYVEm.

### Generation of transgenic parasites

Transgenic parasites were generated by electroporation of 10^7^ tachyzoites with 80 µg plasmid DNA as described [45], and allowed to invade HFFs. The next day, parasites transfected with plasmids p-ddFYVE or p-ddFYVEm were selected with chloramphenicol for three passages, before cloning by limiting dilution under drug selection. Stable transgenic clones expressing both ddFYVE and FNR-RFP were obtained by co-transfection using plasmids FNR-RFP and pminiHXGPRT and selection with 25 µg/ml mycophenolic acid and 50 µg/ml xanthine. Stable transgenic clones expressing ddFYVE/V5-FtsH1 or ddFYVE/APT1 were obtained by transfection using plasmid 2V5FtsH1 [33] and APT1-4HA respectively [34], and selection with 25 µg/ml mycophenolic acid and 50 µg/ml xanthine. These clones were subsequently transiently transfected with FNR-RFP, allowed to invade HFFs and processed for immunofluorescence within two days after transfection.

### Quantification of FNR-RFP positive parasites, invasion and growth assays

For invasion assays, wild-type or ddFYVE intracellular parasites were treated or not with 1 µM Shield-1 for 48 h. The infected monolayer was then scraped and syringed through a 26 gauge needle to allow vacuole lysis and liberation of free tachyzoites. These latter (5×10^6^ parasites) were used to infect confluent HFF grown on glass coverslips in the presence or not of 1 µM Shield-1. Cells were fixed after one hour with 4% formaldehyde for 30 min, and invasion was quantified by counting the number of intracellular tachyzoites. For intracellular growth assay, the number of parasites per vacuole was counted 24 hours post-infection in the presence or absence of 1 µM Shield-1. For delayed death assays, wild-type or ddFYVE intracellular parasites were treated or not with 1 µM Shield-1 for 48 h. Parasites were then isolated by syringing and used to infect confluent HFF grown on glass coverslips (5×10^6^ parasites per well) in the presence or absence of 1 µM Shield-1, and the number of parasites per vacuole was counted 24 h later. The number of parasites retaining their apicoplast after 48 h in the presence or absence of Shield-1 was quantified by counting the number of parasites showing FNR-RFP sub-apical labelling.

### Antibodies, Western blotting and immunofluorescence analysis

Western blots and immunofluorescence were performed as described [46] using rabbit anti-HSP60 [47], rat anti-HA antiserum (Covance, Berkeley), anti-PI3P monoclonal antibodies (Echelon), anti-SAG1 1E5 [48], anti-V5 monoclonal antibodies (Invitrogen), and anti-ROP2/3/4 T3 4A7 [49], anti-MIC2 T34A11[50], anti-ATrx1 11G8 [32] and anti-F1-ATPase beta subunit 5F4 (P. Bradley, unpublished) monoclonal antibodies. Briefly, for IFAs of intracellular parasites, infected confluent HFF monolayers were fixed for 20 min in 4% paraformaldehyde in PBS, permeabilized with 0.2% triton X-100, blocked with 10% FCS in PBS, and then incubated with primary antibodies. Alexa 594- and/or Alexa 488- (Sigma) and Alexa 647- (Invitrogen) conjugated secondary antibodies were used. Coverslips were mounted onto microscope slides using Immumount (Calbiochem). Images were collected either i) with a Leica DMRA2 microscope equipped for epifluorescence, the images being recorded with a COOLSNAP CCD camera (Photometrics) driven by the Metaview software (Universal Imaging Co.) or ii) with a Zeiss Axioimager epifluorescence microscope and images were recorded with a Zeiss Axiocam MRm CCD camera driven by the Axiovision software (Zeiss), at the Montpellier RIO imaging facility. The Zeiss Axioimager epifluorescence microscope was equipped with Apotome illumination enabling the capture of single plane optical sections.

Quantitative analysis of FNR-RFP and ddFYVE-GFP co-localization was performed using the JaCoP plug-in program [51] of the ImageJ software (NIH), and the Manders co-localization coefficients were compared between both channels.

### Live video-microscopy

Live video-microscopy was performed on cells plated on 35 mm glass-bottom Petri dishes, infected and incubated in complete culture medium supplemented with 50 mM Hepes, pH 7.3. They were mounted on a DMIRE2 Leica inverted microscope equipped with an incubation chamber maintained at 37°C and fitted with a Coolsnap Fx cooled CCD camera (Princeton Instruments, Roper Scientific, Evry, France) and driven by the Metamorph software (Universal Imaging, Roper Scientific). Z-stacks of 3 µm were acquired in trans and epi-fluorescence illumination at a 10 min acquisition lapse using a 100x/1.40 oil immersion objective coupled to a piezo device operated with a 1 µm step to select the appropriate focus plane.

### Electron microscopy

Infected monolayers were fixed with 4% paraformaldehyde in 0.2 M Na-phosphate buffer pH 7.4 for 90 min, then washed in PBS-10%FCS (FCS-Sigma), scraped and embedded as a pellet in 5% bovine skin gelatin in PBS-FCS, and infused in 2.3 M sucrose containing 10% polyvinylpyrrolidone before being frozen in liquid nitrogen. Sections were obtained on a Leica Ultracut E equiped with a FCS cryo-attachment operating at –100°C. Sections were floated successively on PBS-FCS, rabbit anti-GFP antibodies (Abcam) diluted 1∶200 in PBS-FCS, 10 nm Protein A-gold (Cell Microscopy Center, University of Utrecht) diluted in PBS to 0.05 OD_525_, with 5×3 min washes in PBS between each step. Sections were then embedded in methylcellulose (2%)-uranyle acetate (0.4%) and observed with a ZEISS EM10 electron microscope. For double immuno-EM, the first round of detection was followed by washing with PBS-FCS, 2 min fixation with 1% glutaraldehyde in PBS, followed by mouse antibody against V5 or HA tag, rabbit antimouse and 5 nm protein A-gold. Single detections with each of the antibodies were also performed to control their specificity.

## Supporting Information

Figure S1 Parasites transiently transfected with GFP-2xFYVE were analysed by time lapse fluorescence microscopy. Shown are selected images of Video S1 (time indicated in hours). The entire apical GFP label collapses into the already strongly labelled residual body (arrowhead) during cell division and new PI3P-containing compartments rapidly form in the nascent daughter cells (arrows). Multiple fluorescence signals in one parasite (see 5 h) finally fuse at the apical site (see 7 h).
(0.42 MB PPT)

Figure S2 HFF monolayers infected for 24 h with wild-type parasites were fixed in 4% paraformaldehyde, permeabilized with 0.1% Triton X-100, labelled with anti-PI3P antibodies (1∶150) and either with the DNA stain Hoechst 33342 to detect the apicoplast (A) or with anti-HSP60, a protein stored in the stroma of the apicoplast (B). Images were taken using a Zeiss Axioimager microscope fitted with an apotome illumination. Single plane apotome sections show the presence of PI3P (green) at the apicoplast that is identified by its DNA stained as a small dot next to the nucleus (colored in red) (A), or by a partial co-localization with HSP60 (B).
(0.50 MB PPT)

Figure S3 Quantitative analysis of FNR-RFP/ddFYVE co-localization before and 4 h after Shield-1 addition. HFF monolayers infected for 24 h with the ddFYVE/FRN-RFP transfected parasites were fixed and observed with a Zeiss Axioimager microscope fitted with an apotome illumination and using a 63× apochromat objective (n.a. 1.4). Red and green fluorescence images of Z sections and DIC images were recorded sequentially using the Zeiss Axiocam MRm CCD camera driven by the Axiovision software. The red and green signals of 10 representative vacuoles for each time point were analyzed using the JaCoP program of ImageJ and the M1 and M2 Manders co-localization coefficients were collected in each case (M1: fraction of the FNR-RFP signal overlapping the ddFYVE-GFP signal; M2: fraction of the GFP overlapping the RFP; the Manders coefficient value ranges from 0 to 1 corresponding to no overlap and to full overlap, respectively). The mean and standard deviation for both coefficients were calculated for t = 0 and t = 4 h. Image series T0 #5 and T4h #8 are shown to illustrate the co-localization difference found between T0 and T4h and quantified as described above. Scale bar  = 2 µm.
(0.44 MB PPT)

Figure S4 Over-expression of ddFYVE did not disturb the localization of rhoptry-, microneme-, Golgi-, mitochondrion-, and endosome compartment markers. Intracellular parasites expressing ddFYVE were incubated with 1 µM Shield-1 for 4 h, and processed for IFA using antibodies to rhoptry proteins ROP2/3/4 (A) and to microneme protein MIC2 (B). For endosome- and Golgi detection, ddFYVE parasites were co-transfected with plasmids allowing expression of either HA-tagged Rab51 protein (endosome marker, C) or GRASP-RFP protein (Golgi marker, D). Rab51-HA was detected using anti-HA antibodies. For mitochondrion detection (E), intracellular parasites expressing ddFYVE and FNR-RFP were incubated with 1 µM Shield-1 for 4 h and labelled with the antibodies against mitochondrial F1-ATPase. Scale bar  = 2 µm.
(0.56 MB PPT)

Figure S5 The PI3-kinase inhibitor LY294002 did not disturb the localization of rhoptry, microneme, mitochondrion and endosome compartment markers. Stable FNR-RFP transfected parasites were incubated with 100 µM LY294002 for 4 h or mock treated (A, B, C). While the treatment led to severe disturbance of the FNR-RFP apicoplast label with several parasites having lost the organelle, it did not affect the localization of the rhoptry marker ROP2/3/4 (A), the microneme marker MIC2 (B) and the endosome marker Rab51 (C). MIC2 and ROP2/3/4 were detected using specific antibodies, while Rab51 was localised by co-transfection with HA-Rab51 and immuno-localization using anti-HA antibodies. (D) For analysis of the mitochondrion, ddFYVE/FRN-RFP expressing parasites were treated with 100 µM LY294002 for 4 h or mock treated before detection of PI3P by ddFYVE stabilisation for 20 min with 1 µM Shield-1. The mitochondrion was labelled with the anti-F1-ATPase. Scale bar  = 2 µm.
(0.81 MB PPT)

Figure S6 Morphological changes of the apicoplast after LY294002 treatment. Intracellular parasites were treated with various concentration of LY294002 for 3 hours and the apicoplast was visualized by IFA using the luminal marker HSP60 and the nucleus by Hoechst 33342 staining. One hundred vacuoles were counted for each concentration of LY294002. The vacuoles were classified into five different types that are depicted schematically: in grey, the outline of the parasite; in red, the shapes of the apicoplast (compact, V-shape elongated, enlarged or lost); in blue, the nucleus. Included in the five groups are all the vacuoles that contained at least one parasite without apicoplast.
(0.20 MB PPT)

Figure S7 Electron microscopy analysis of RH parasites fixed 4 h after 100 µM LY294002 addition. (A) Parasites in this vacuole show swollen apicoplasts (a) with internal myelinic profiles and one apicoplast that has been discarded into the residual body (a1). Bar: 1 µm. (B) Enlargement of an adjacent section of the a1 apicoplast shown in A. Bar: 0.2 µm.
(5.43 MB TIF)

Figure S8 The traffic of outermost apicoplast membrane proteins is disturbed in the presence of ddFYVE. Immunofluorescence analysis of ddFYVE/FNR-RFP/V5-FtsH1 or ddFYVE/FNR-RFP/APT1-HA triple transfected parasites treated for 24 h with Shield-1 revealed a more diffuse labelling of FtsH1 and APT1 surrounding the luminal apicoplast marker FNR as compared to untreated parasites. The merged images only show FNR and FtsH1 or APT1, respectively.
(1.78 MB PPT)

Video S1 Parasites transiently transfected with GFP-2xFYVE were analysed by time lapse fluorescence microscopy. Data acquisition was started 18 h after transfection and one image taken every 15 min. The video shows the collapse of the entire apical GFP label into the already strongly labelled residual body during cell division and the immediate formation of new PI3P-containing compartments in the nascent daughter cells.
(3.42 MB AVI)

Video S2 The parasite clone stably expressing ddFYVE was transfected with FNR-RFP and grown intracellularly without Shield-1 for 18 hours. Data acquisition was started 2 h after addition of 1 µM Shield-1 and images were taken every 10 minutes. The video shows an overlay of phase contrast, ddFYVE (green) and FNR-RFP (red) images. The vacuole of interest containing four parasites co-expressing both ddFYVE and FNR-RFP is located in the upper right corner at the beginning of the video. During apicoplast elongation, the FNR signal surrounds the GFP label and there is no GFP staining in the residual body (t = 120–200 min). Later, the FNR signal elongates further and moves away from GFP (t = 240 min). Seven small distinct FNR spots (one slightly out of focus) in the sub-apical part correspond to the apicoplasts of the newly formed daughter cells, the eighth one having lost the label. Additional FNR spots are found close to or co-localized with a large GFP signal that ultimately accumulates in the residual body (t = 300 min). The newly apicoplasts in the newly formed daughter cells rapidly became GFP positive (not visible in the merged images). Selected images of the vacuole of interest are depicted in Figure 2B.
(7.82 MB AVI)

Video S3 The same acquisition as in Video S1 is presented showing only the green ddFYVE- and the red FNR label, allowing clearer visualisation of the PI3P-containing compartment and of the apicoplast.
(7.82 MB AVI)
