# Supplementary figures and images for: Phosphatidylinositol 3-Monophosphate Is Involved in Toxoplasma Apicoplast Biogenesis
Source: PLoS Pathog. 2011 Feb 17;7(2):e1001286. doi: 10.1371/journal.ppat.1001286 (PMC3040667; doi:10.1371/journal.ppat.1001286)

## Slide 1
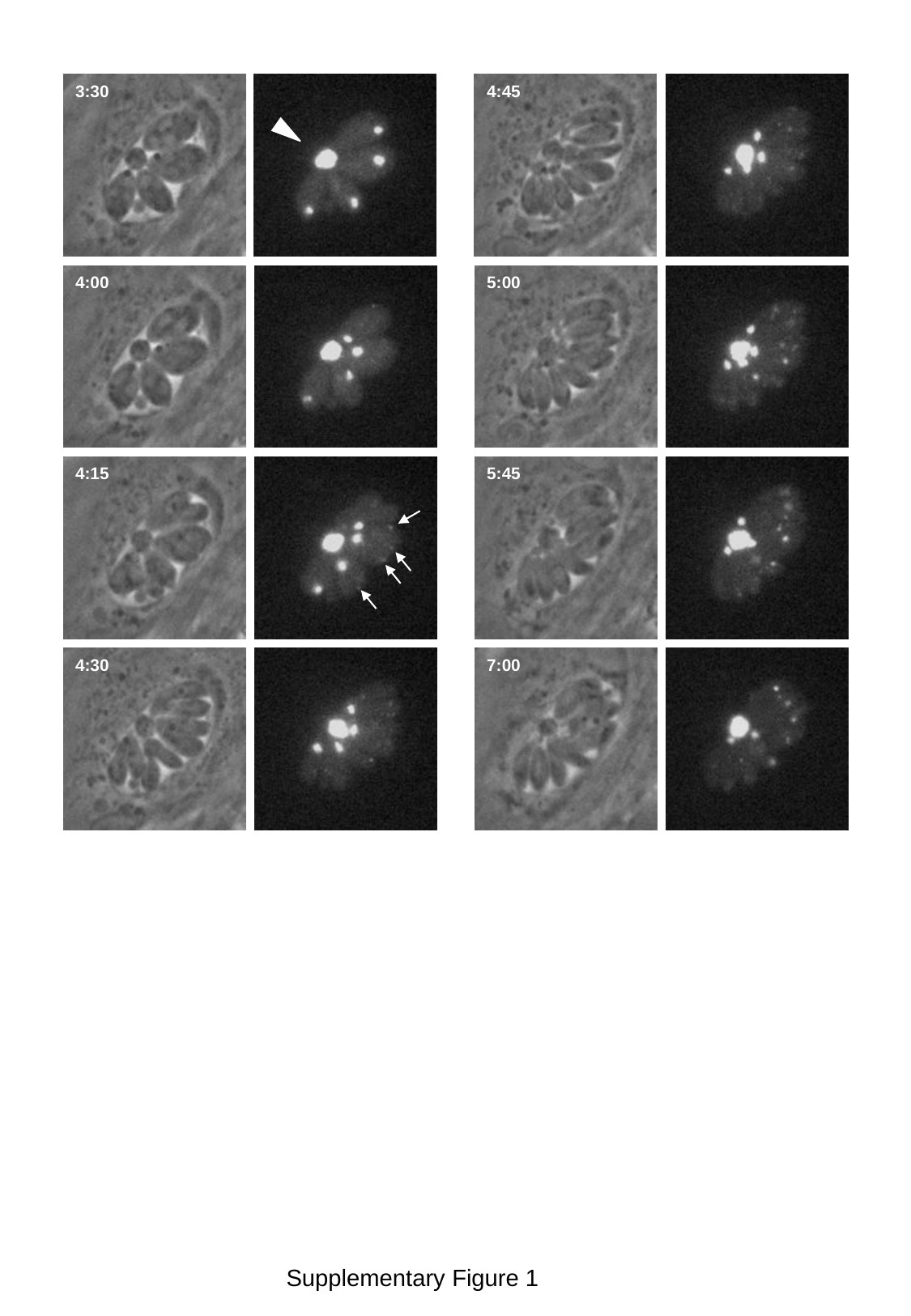

3:30
4:45
4:00
5:00
4:15
5:45
4:30
7:00
Supplementary Figure 1

Supplement: Figure S1 — Parasites transiently transfected with GFP-2xFYVE were analysed by time lapse fluorescence microscopy. Shown are selected images of Video S1 (time indicated in hours). The entire apical GFP label collapses into the already strongly labelled residual body (arrowhead) during cell division and new PI3P-containing compartments rapidly form in the nascent daughter cells (arrows). Multiple fluorescence signals in one parasite (see 5 h) finally fuse at the apical site (see 7 h). (0.42 MB PPT) [file ppat.1001286.s001.ppt]

## Slide 1
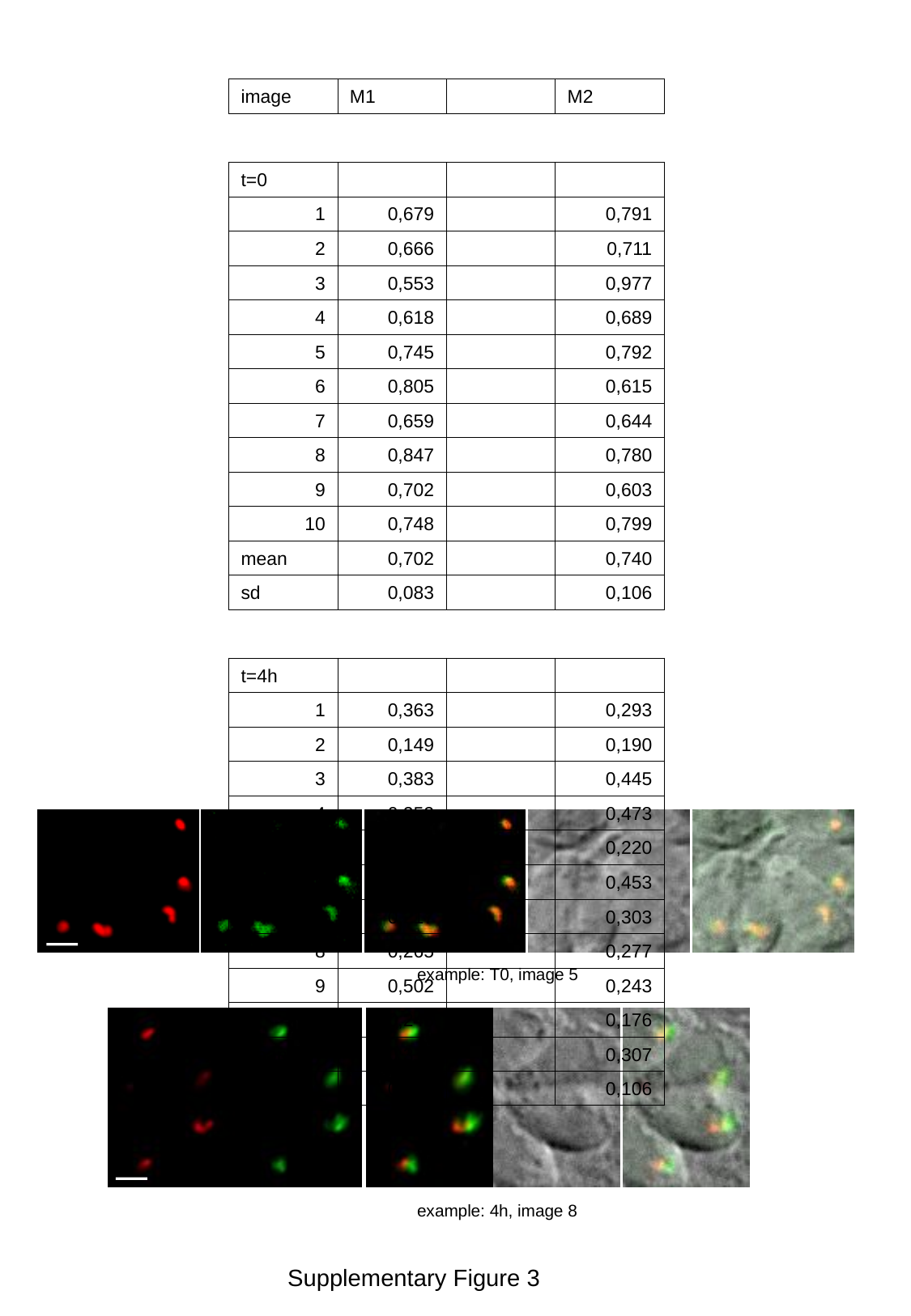

| image | M1 | | M2 |
| --- | --- | --- | --- |
| | | | |
| t=0 | | | |
| 1 | 0,679 | | 0,791 |
| 2 | 0,666 | | 0,711 |
| 3 | 0,553 | | 0,977 |
| 4 | 0,618 | | 0,689 |
| 5 | 0,745 | | 0,792 |
| 6 | 0,805 | | 0,615 |
| 7 | 0,659 | | 0,644 |
| 8 | 0,847 | | 0,780 |
| 9 | 0,702 | | 0,603 |
| 10 | 0,748 | | 0,799 |
| mean | 0,702 | | 0,740 |
| sd | 0,083 | | 0,106 |
| | | | |
| t=4h | | | |
| 1 | 0,363 | | 0,293 |
| 2 | 0,149 | | 0,190 |
| 3 | 0,383 | | 0,445 |
| 4 | 0,252 | | 0,473 |
| 5 | 0,390 | | 0,220 |
| 6 | 0,390 | | 0,453 |
| 7 | 0,217 | | 0,303 |
| 8 | 0,265 | | 0,277 |
| 9 | 0,502 | | 0,243 |
| 10 | 0,245 | | 0,176 |
| mean | 0,316 | | 0,307 |
| sd | 0,101 | | 0,106 |
| example: T0, image 5 |
| --- |
| example: 4h, image 8 |
| --- |
Supplementary Figure 3

Supplement: Figure S3 — Quantitative analysis of FNR-RFP/ddFYVE co-localization before and 4 h after Shield-1 addition. HFF monolayers infected for 24 h with the ddFYVE/FRN-RFP transfected parasites were fixed and observed with a Zeiss Axioimager microscope fitted with an apotome illumination and using a 63× apochromat objective (n.a. 1.4). Red and green fluorescence images of Z sections and DIC images were recorded sequentially using the Zeiss Axiocam MRm CCD camera driven by the Axiovision software. The red and green signals of 10 representative vacuoles for each time point were analyzed using the JaCoP program of ImageJ and the M1 and M2 Manders co-localization coefficients were collected in each case (M1: fraction of the FNR-RFP signal overlapping the ddFYVE-GFP signal; M2: fraction of the GFP overlapping the RFP; the Manders coefficient value ranges from 0 to 1 corresponding to no overlap and to full overlap, respectively). The mean and standard deviation for both coefficients were calculated for t = 0 and t = 4 h. Image series T0 #5 and T4h #8 are shown to illustrate the co-localization difference found between T0 and T4h and quantified as described above. Scale bar = 2 µm. (0.44 MB PPT) [file ppat.1001286.s003.ppt]

## Slide 1
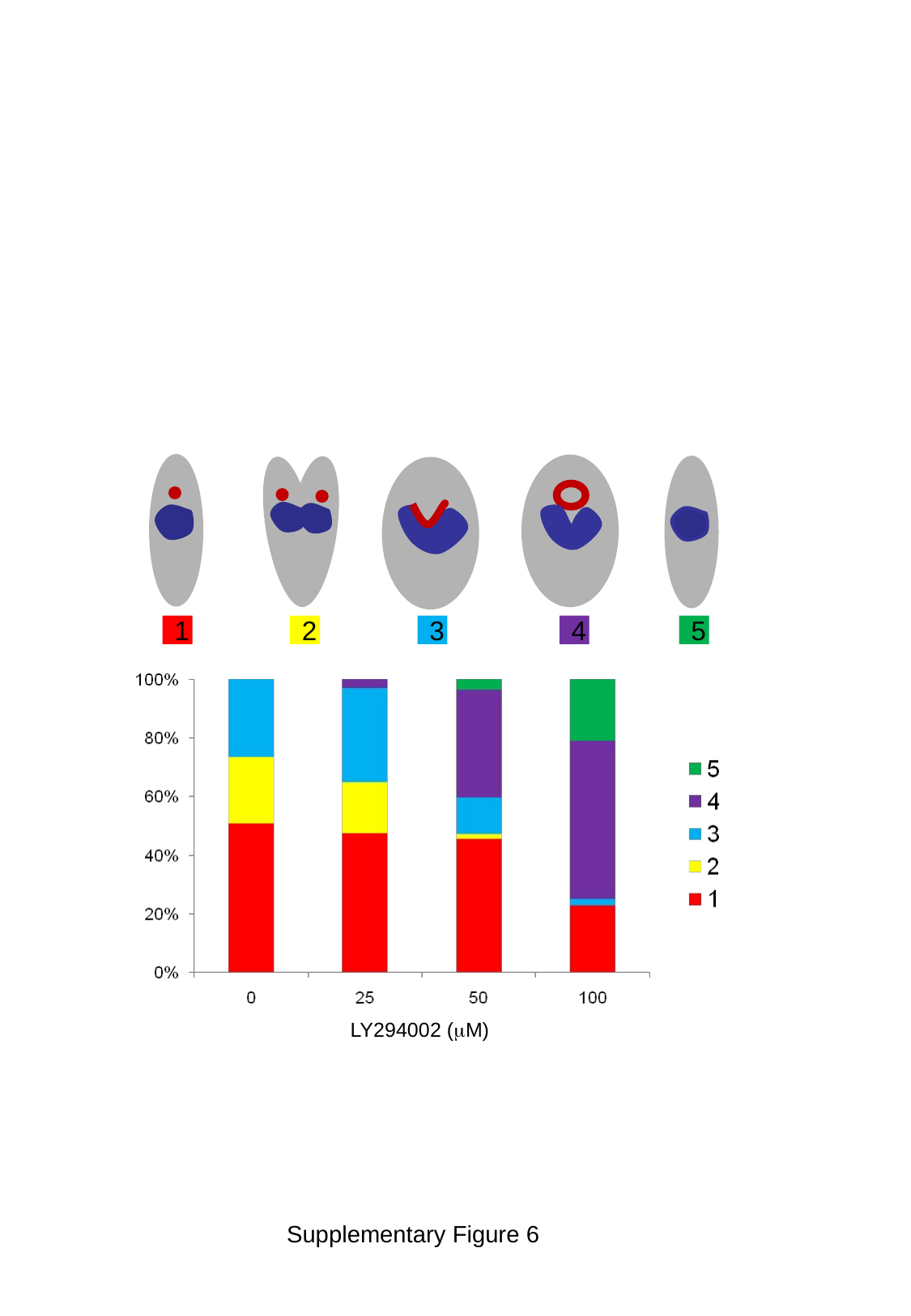

1
2
3
4
5
LY294002 (μM)
Supplementary Figure 6

Supplement: Figure S6 — Morphological changes of the apicoplast after LY294002 treatment. Intracellular parasites were treated with various concentration of LY294002 for 3 hours and the apicoplast was visualized by IFA using the luminal marker HSP60 and the nucleus by Hoechst 33342 staining. One hundred vacuoles were counted for each concentration of LY294002. The vacuoles were classified into five different types that are depicted schematically: in grey, the outline of the parasite; in red, the shapes of the apicoplast (compact, V-shape elongated, enlarged or lost); in blue, the nucleus. Included in the five groups are all the vacuoles that contained at least one parasite without apicoplast. (0.20 MB PPT) [file ppat.1001286.s006.ppt]

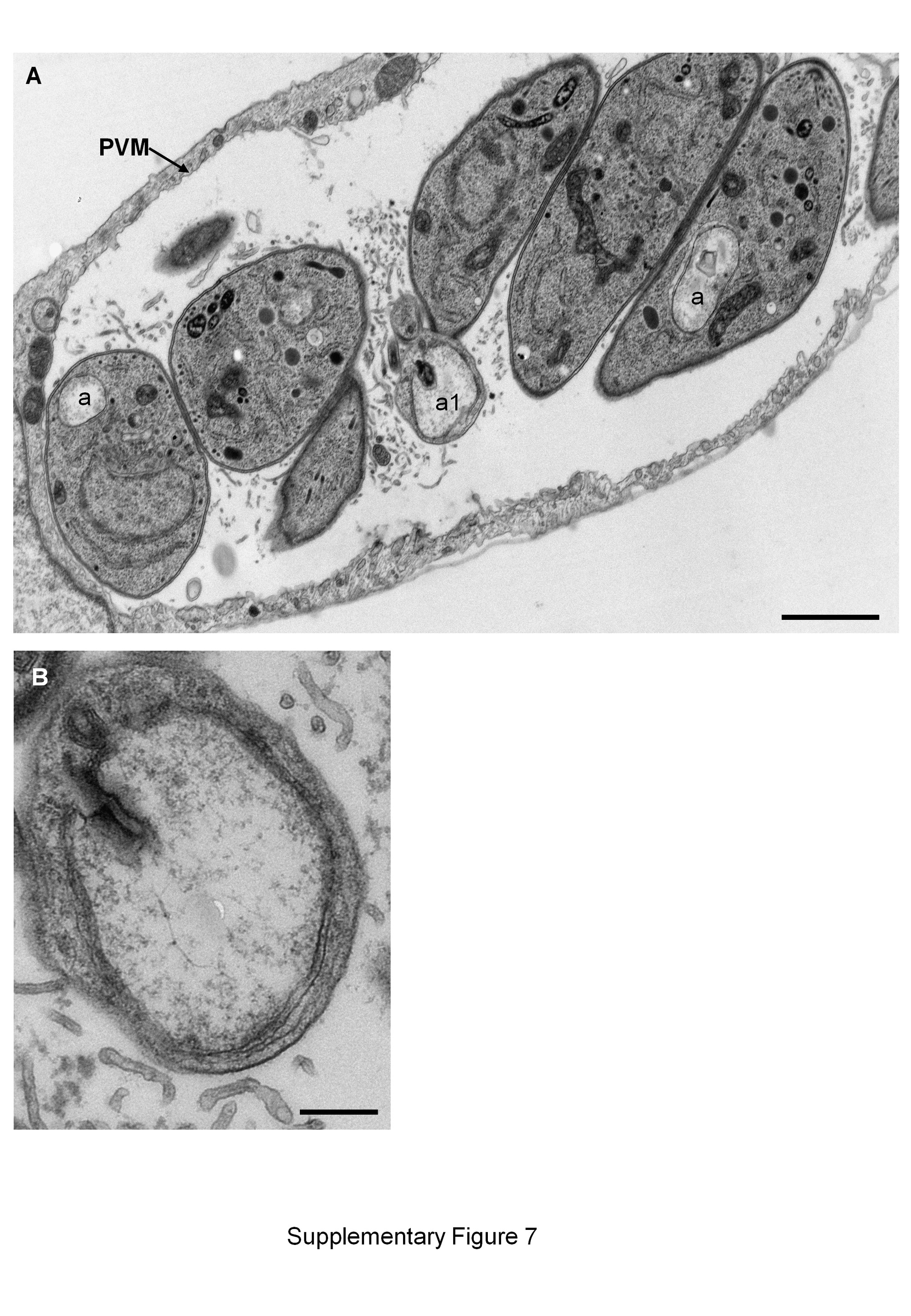

Supplement: Figure S7 — Electron microscopy analysis of RH parasites fixed 4 h after 100 µM LY294002 addition. (A) Parasites in this vacuole show swollen apicoplasts (a) with internal myelinic profiles and one apicoplast that has been discarded into the residual body (a1). Bar: 1 µm. (B) Enlargement of an adjacent section of the a1 apicoplast shown in A. Bar: 0.2 µm. (5.43 MB TIF) [file ppat.1001286.s007.tif]
