# Supplementary material for: Phosphatidylinositol 3-Monophosphate Is Involved in Toxoplasma Apicoplast Biogenesis
Source: PLoS Pathog. 2011 Feb 17;7(2):e1001286. doi: 10.1371/journal.ppat.1001286 (PMC3040667; doi:10.1371/journal.ppat.1001286)
Supplement: Figure S2 — HFF monolayers infected for 24 h with wild-type parasites were fixed in 4% paraformaldehyde, permeabilized with 0.1% Triton X-100, labelled with anti-PI3P antibodies (1∶150) and either with the DNA stain Hoechst 33342 to detect the apicoplast (A) or with anti-HSP60, a protein stored in the stroma of the apicoplast (B). Images were taken using a Zeiss Axioimager microscope fitted with an apotome illumination. Single plane apotome sections show the presence of PI3P (green) at the apicoplast that is identified by its DNA stained as a small dot next to the nucleus (colored in red) (A), or by a partial co-localization with HSP60 (B). (0.50 MB PPT) [file ppat.1001286.s002.ppt]

## Slide 1
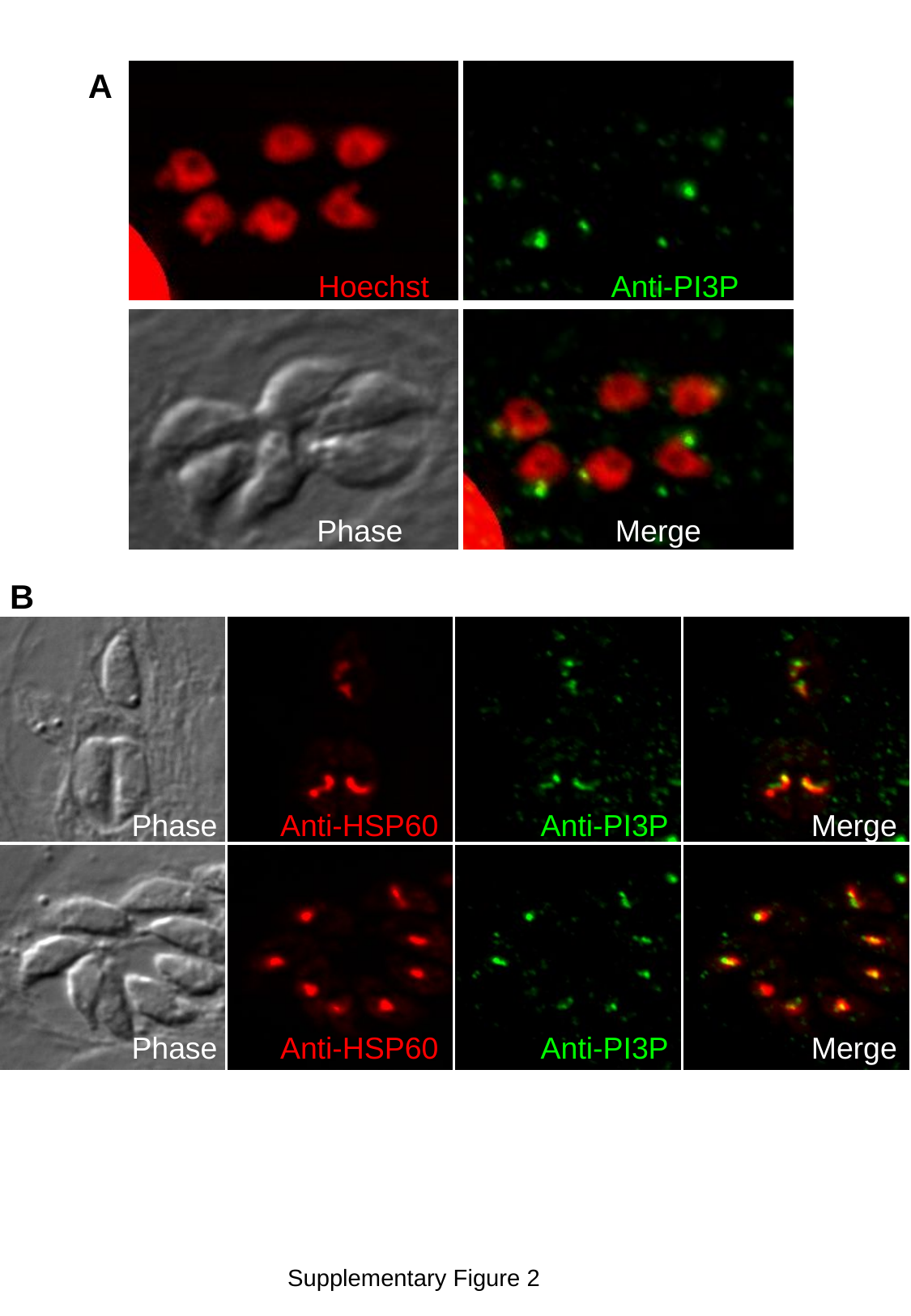

A
Hoechst
Anti-PI3P
Phase
Merge
B
Phase
Anti-HSP60
Anti-PI3P
Merge
Phase
Anti-HSP60
Anti-PI3P
Merge
Supplementary Figure 2
