# Supplementary material for: Phosphatidylinositol 3-Monophosphate Is Involved in Toxoplasma Apicoplast Biogenesis
Source: PLoS Pathog. 2011 Feb 17;7(2):e1001286. doi: 10.1371/journal.ppat.1001286 (PMC3040667; doi:10.1371/journal.ppat.1001286)
Supplement: Figure S4 — Over-expression of ddFYVE did not disturb the localization of rhoptry-, microneme-, Golgi-, mitochondrion-, and endosome compartment markers. Intracellular parasites expressing ddFYVE were incubated with 1 µM Shield-1 for 4 h, and processed for IFA using antibodies to rhoptry proteins ROP2/3/4 (A) and to microneme protein MIC2 (B). For endosome- and Golgi detection, ddFYVE parasites were co-transfected with plasmids allowing expression of either HA-tagged Rab51 protein (endosome marker, C) or GRASP-RFP protein (Golgi marker, D). Rab51-HA was detected using anti-HA antibodies. For mitochondrion detection (E), intracellular parasites expressing ddFYVE and FNR-RFP were incubated with 1 µM Shield-1 for 4 h and labelled with the antibodies against mitochondrial F1-ATPase. Scale bar = 2 µm. (0.56 MB PPT) [file ppat.1001286.s004.ppt]

## Slide 1
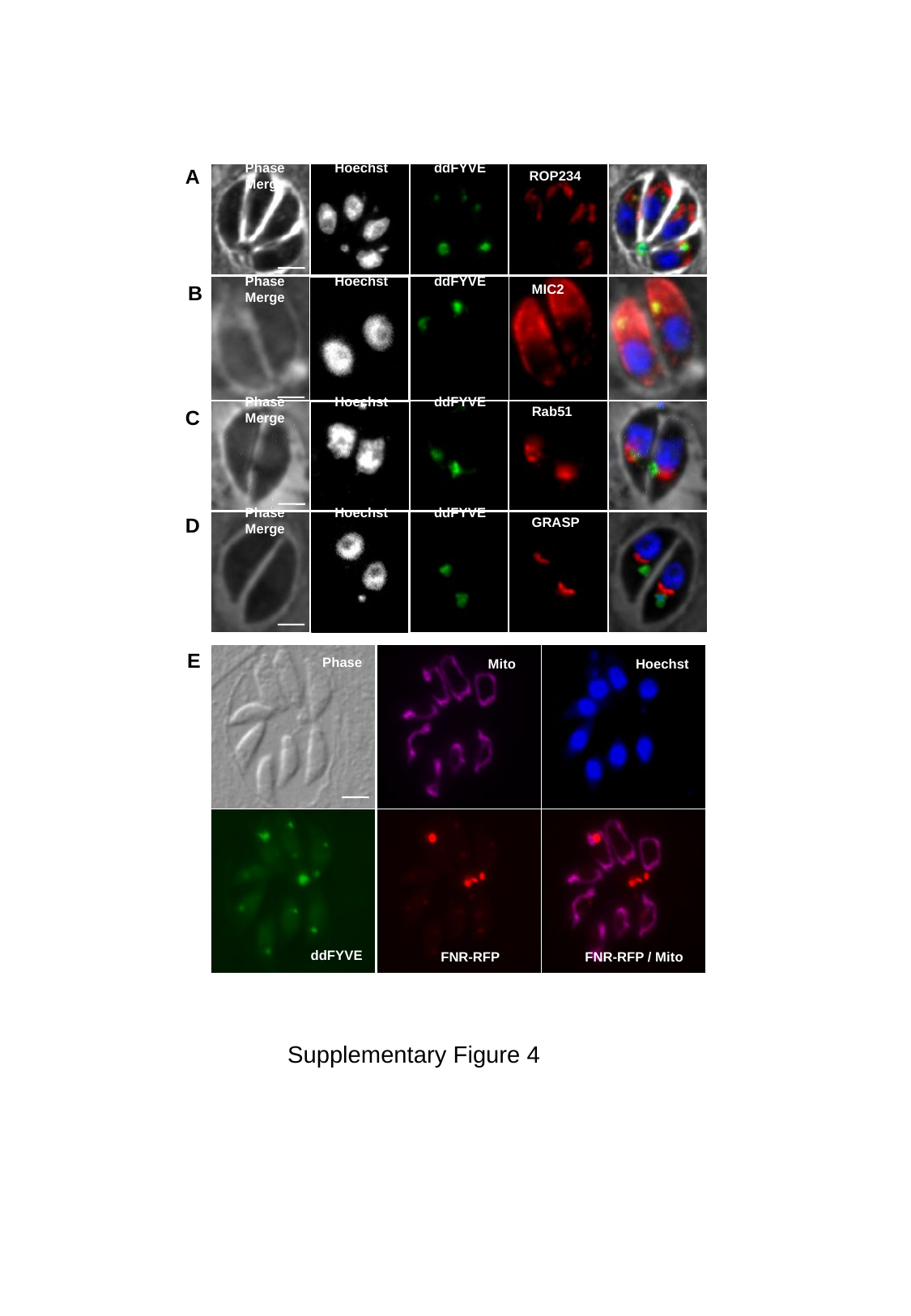

Phase Hoechst ddFYVE Merge
A
ROP234
Phase Hoechst ddFYVE Merge
B
MIC2
Phase Hoechst ddFYVE Merge
Rab51
C
Phase Hoechst ddFYVE Merge
D
GRASP
E
Phase
Mito
Hoechst
ddFYVE
FNR-RFP
FNR-RFP / Mito
Supplementary Figure 4
