# Supplementary material for: Phosphatidylinositol 3-Monophosphate Is Involved in Toxoplasma Apicoplast Biogenesis
Source: PLoS Pathog. 2011 Feb 17;7(2):e1001286. doi: 10.1371/journal.ppat.1001286 (PMC3040667; doi:10.1371/journal.ppat.1001286)
Supplement: Figure S5 — The PI3-kinase inhibitor LY294002 did not disturb the localization of rhoptry, microneme, mitochondrion and endosome compartment markers. Stable FNR-RFP transfected parasites were incubated with 100 µM LY294002 for 4 h or mock treated (A, B, C). While the treatment led to severe disturbance of the FNR-RFP apicoplast label with several parasites having lost the organelle, it did not affect the localization of the rhoptry marker ROP2/3/4 (A), the microneme marker MIC2 (B) and the endosome marker Rab51 (C). MIC2 and ROP2/3/4 were detected using specific antibodies, while Rab51 was localised by co-transfection with HA-Rab51 and immuno-localization using anti-HA antibodies. (D) For analysis of the mitochondrion, ddFYVE/FRN-RFP expressing parasites were treated with 100 µM LY294002 for 4 h or mock treated before detection of PI3P by ddFYVE stabilisation for 20 min with 1 µM Shield-1. The mitochondrion was labelled with the anti-F1-ATPase. Scale bar = 2 µm. (0.81 MB PPT) [file ppat.1001286.s005.ppt]

## Slide 1
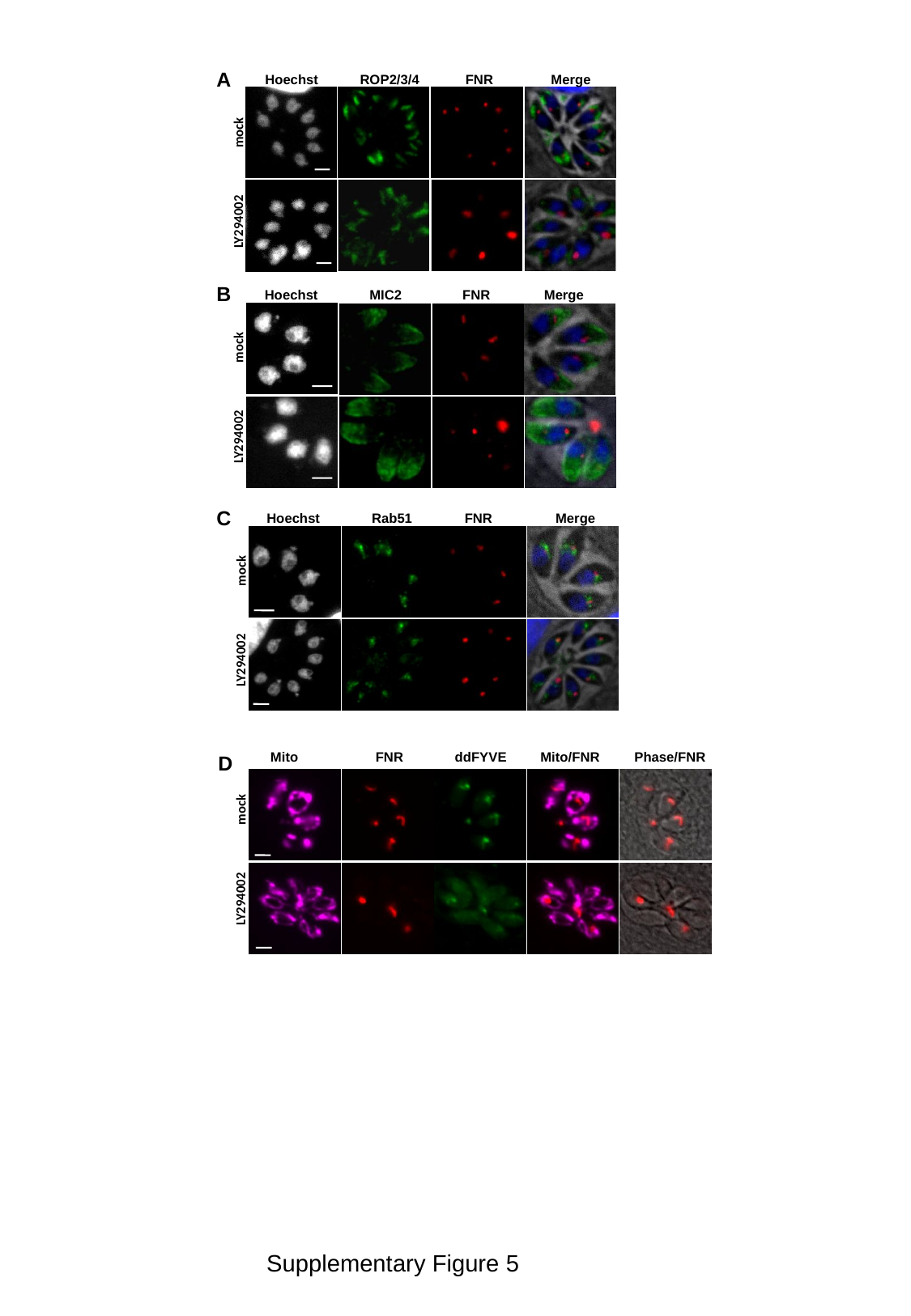

A
Hoechst
ROP2/3/4
FNR
Merge
mock
LY294002
B
Hoechst
MIC2
FNR
Merge
mock
LY294002
C
Hoechst
Rab51
FNR
Merge
mock
LY294002
Mito
FNR
ddFYVE
Mito/FNR Phase/FNR
D
mock
LY294002
Supplementary Figure 5
