# Supplementary material for: Phosphatidylinositol 3-Monophosphate Is Involved in Toxoplasma Apicoplast Biogenesis
Source: PLoS Pathog. 2011 Feb 17;7(2):e1001286. doi: 10.1371/journal.ppat.1001286 (PMC3040667; doi:10.1371/journal.ppat.1001286)
Supplement: Figure S8 — The traffic of outermost apicoplast membrane proteins is disturbed in the presence of ddFYVE. Immunofluorescence analysis of ddFYVE/FNR-RFP/V5-FtsH1 or ddFYVE/FNR-RFP/APT1-HA triple transfected parasites treated for 24 h with Shield-1 revealed a more diffuse labelling of FtsH1 and APT1 surrounding the luminal apicoplast marker FNR as compared to untreated parasites. The merged images only show FNR and FtsH1 or APT1, respectively. (1.78 MB PPT) [file ppat.1001286.s008.ppt]

## Slide 1
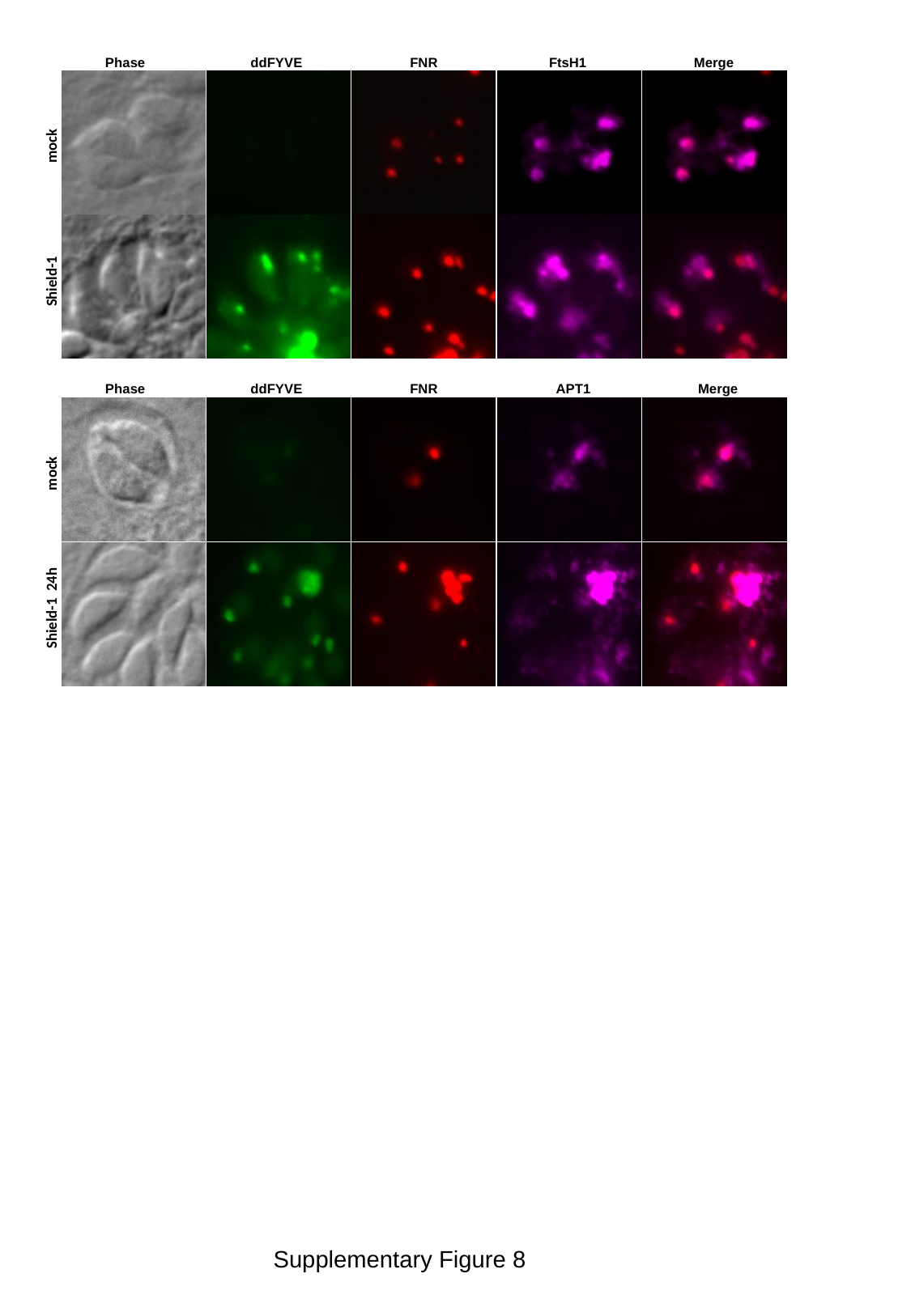

FNR FtsH1 Merge
Phase
ddFYVE
mock
Shield-1
 FNR APT1 Merge
Phase
ddFYVE
mock
Shield-1 24h
Supplementary Figure 8
